# The association between socioeconomic status and pandemic influenza: Systematic review and meta-analysis

**DOI:** 10.1371/journal.pone.0244346

**Published:** 2021-09-07

**Authors:** Svenn-Erik Mamelund, Clare Shelley-Egan, Ole Rogeberg

**Affiliations:** 1 Centre for Research on Pandemics & Society, Oslo Metropolitan University, Oslo, Norway; 2 Work Research Institute, Oslo Metropolitan University, Oslo, Norway; 3 Frisch Centre, University of Oslo, Oslo, Norway; Nnamdi Azikiwe University, NIGERIA

## Abstract

**Background:**

The objective of this study is to document whether and to what extent there is an association between socioeconomic status (SES) and disease outcomes in the last five influenza pandemics.

**Methods/principle findings:**

The review included studies published in English, Danish, Norwegian and Swedish. Records were identified through systematic literature searches in six databases. We summarized results narratively and through meta-analytic strategies. Only studies for the 1918 and 2009 pandemics were identified. Of 14 studies on the 2009 pandemic including data on both medical and social risk factors, after controlling for medical risk factors 8 demonstrated independent impact of SES. In the random effect analysis of 46 estimates from 35 studies we found a pooled mean odds ratio of 1.4 (95% CI: 1.2–1.7, p < 0.001), comparing the lowest to the highest SES, but with substantial effect heterogeneity across studies,–reflecting differences in outcome measures and definitions of case and control samples. Analyses by pandemic period (1918 or 2009) and by level of SES measure (individual or ecological) indicated no differences along these dimensions. Studies using healthy controls tended to document that low SES was associated with worse influenza outcome, and studies using infected controls find low SES associated with more severe outcomes. A few studies compared severe outcomes (ICU or death) to hospital admissions but these did not find significant SES associations in any direction. Studies with more unusual comparisons (e.g., pandemic vs seasonal influenza, seasonal influenza vs other patient groups) reported no or negative non-significant associations.

**Conclusions/significance:**

We found that SES was significantly associated with pandemic influenza outcomes with people of lower SES having the highest disease burden in both 1918 and 2009. To prepare for future pandemics, we must consider social vulnerability. The protocol for this study has been registered in PROSPERO (ref. no 87922) and has been published Mamelund et al. (2019).

## Introduction

It used to be believed that pandemic and infectious disease risks are the same for all, irrespective of socioeconomic status (SES). But when 61-year old superstar Madonna shared this belief on Instagram on the 23^rd^ of March 2020, calling COVID-19 “the great equalizer” from a milky bath sprinkled with rose-petals [1], fans and others quickly pointed to the disproportionate pandemic burden and suffering of the poor. Indeed, their criticism is supported by a number of studies showing that certain indigenous people, people of colour, immigrants and the poor have experienced disproportionate harm as a result of COVID-19 as measured by infection rates, hospitalizations, the need for intensive care unit treatment, and death [2–5].

The idea that outcomes from infectious disease pandemics are socially neutral has a long history among lay people, researchers and policy makers responsible for pandemic preparedness plans. Literature on SES and 1918 influenza outcomes published by social historians between 1970 and 1990 argued that the disease was so highly transmissible that everybody was equally affected [6–10], pointing to anecdotal evidence such as the president and King of Spain falling ill and the Swedish Prince Erik dying at age 29 [11]. However, these studies used aggregate-level data, were mainly descriptive and did not use multivariate statistical models. Empirical studies appearing from the mid-2000s often reported evidence inconsistent with the socially neutral hypothesis: SES seemed to be linked to exposure, susceptibility and access to care, and SES indicators were statistically associated with mortality [12–14]. Although several studies of the 2009 pandemic also found SES associated with various pandemic outcomes [15–17], this social inequality in risk is still ignored in international influenza pandemic preparedness plans [18]. Apart from a systematic review and meta-analysis of the 2009 pandemic disease burden in low and low to middle income economies and differences in disease outcomes in that pandemic for ethnic minorities vs non-ethnic minorities [19], a systematic assessment of several influenza pandemics and of the evidence for disparities in pandemic outcomes by individual and/or area-level SES (e.g. education, income, household crowding and quality, unemployment, occupation-based social class, poverty status, share below poverty levels, deprivation indexes etc.) has been lacking.

In this paper, we present the first systematic review and meta-analysis on the association between SES and disease outcomes in the last 5 influenza pandemics. The objective is to document whether and to what extent there is an association between indicators of socioeconomic status (e.g. income, education) and pandemic outcomes (infection, hospitalizations, mortality) in the last five influenza pandemics (1889, 1918, 1957, 1968, 2009). In terms of PICOS criteria, the Population (P) consists of groups defined by socioeconomic status, the intervention (I) or exposure or risk factor is pandemic influenza, the comparison (C) or alternative interventions is not relevant, while the outcomes (O) are morbidity, hospitalization, or death associated with influenza pandemics. All types of study design were considered (S). As described in our pre-registered analysis plan, we hypothesized that the association between SES and pandemic outcomes would increase with outcome severity, as higher income and SES tend to be associated with access to resources and protective factors that reduce the risk of progression to more severe outcomes.

## Materials and methods

### Bibliographic database search

A systematic search of Medline, Embase, Cinahl, SocIndex, Scopus and Web of Science was performed to identify all relevant articles published on SES and pandemic influenza (morbidity, severe disease and mortality). SES was captured by keywords such as education, income, occupational social class etc. (see search strategy, S1 Table, for more examples). Morbidity was captured by keywords such as infection rates, transmission rates, lab confirmed influenza, flu like illness, and influenza like illness (ILI). Severe disease was captured by keywords such as disease severity, critical illness, critical disease, severe illness, severe disease, hospitalization, patient admission, hospital admission, intensive care unit (ICU) admission, and ICU treatment. Mortality was captured by keywords such as fatal outcome, fatal illness, fatal disease, fatality, lethal outcome, lethal illness, lethal disease, terminal outcome, terminal illness, terminal disease, lethality, death, death rate, and mortality rate. All of these keywords were used in both pilots and the final searches. The strategy for the literature search was developed by two information specialists in cooperation with the research group, starting 5 October 2017. Several pilot searches were conducted in Web of Science and Medline respectively, on 12 and 19 October 2017, to ensure a sensitive search. The search strategy combined relevant terms, both controlled vocabulary terms (i.e. MeSH) and text words. The main search strategy used in Medline is available in PROSPERO 87922 and in S1 Table. The final search was carried out on 17 November 2017. The strategy was modified to fit the other databases listed above. To generate manageable results, restrictions on language (English, Danish, Norwegian and Swedish) and publication type (article/research article) were added to the searches in the other databases. The searches in Medline and Embase were performed without publication type restrictions. The search strategy was peer-reviewed by a third information specialist using a structured tool based on the PRESS-framework [20]. Reference lists of relevant known studies were also screened and experts in the field consulted in order to identify other additional sources. Finally, we also contacted authors of published studies to ask for relevant data not presented in the papers or in appendices. However, we did not get any responses to these requests.

### Inclusion criteria for title and abstract screening

After adding all identified records to an Endnote library and removing duplicates, the remaining results were imported to the program Covidence. Here, additional duplicates were removed. The title and abstract of each article was screened by two of the authors (SEM and CSE), according to the selection criteria. After screening of titles and abstracts, we added full-text versions of articles in Covidence. Divergences in the inclusion of studies were re-assessed by the same researchers until consensus was reached in terms of inclusion or exclusion. The criteria for inclusion were:

1. The study period 1889–2009 includes the five pandemics in 1889, 1918, 1957, 1968 and 2009
2. Studies investigating the association between SES and pandemic outcomes
3. Studies of race, ethnicity, and indigenous people that reported data on SES controls
4. Studies addressing both seasonal and pandemic influenza distinguishing between non-pandemic and pandemic years
5. Studies addressing all regions/countries, type of studies (interventional, observational, etc.) and populations (age, gender, pregnant women, soldiers etc.)

### Exclusion criteria for title and abstract screening

The following criteria excluded studies from the systematic literature review:

1. Studies on pandemic diseases other than influenza
2. Studies on seasonal influenza only
3. Studies on both seasonal and pandemic influenza that *did not* distinguish between non-pandemic and pandemic years
4. Studies on attitudes and compliance with (non)pharmaceutical pandemic interventions
5. Qualitative studies on the associations between SES and pandemic outcomes
6. Studies on social justice and pandemic influenza
7. Studies of pandemic influenza preparedness plans
8. Studies of race, ethnicity, and indigenous people that *did not* report data on SES controls

### Data selection and extraction

We drafted a data abstraction form, pilot-tested it and modified it, when necessary. Two reviewers (SEM and CSE) independently extracted data from all included studies. Any disagreements were resolved via discussion or by involving a third reviewer for arbitration. 1–5 and 6 below were entered into separate spreadsheets for each article. The following information was extracted:

1. Article info
  - First author
  - Year published
  - Journal
2. Data sample
  - Country or region of analysis
  - Pandemic years (1889, 1918, 1957, 1968, 2009)
  - Sample inclusion criteria–i.e. characteristics of sample/population (civilian, military, gender, pregnant, age-group/median/average age, patient group etc).
  - Sample size
  - Unit of analysis (individuals, households, regions, hospitals etc)
  - Data aggregation level (observations of individual units, aggregated units, etc.). e.g., if hospitals are the unit of analysis, does the data used occur at the hospital level or is it pooled across hospitals?
  - Source of outcome data, e.g., census, routine notification data (e.g. influenza cases reported to a doctor), survey data, register data
    - If survey or population data had incomplete coverage
      1. Response rate/coverage
      2. Representativity: Is the sample shown to be representative for the population? i.e. has a non-response analysis been carried out?
3. Outcome variable—Pandemic outcome (a. morbidity, b. hospitalization, c. mortality)
  - Definition of morbidity: influenza-like illness (ILI), Lab-confirmed Infection rates (PCR), transmission rates (reproduction number, R0), immunity/antibodies towards influenza (HI titer above a certain threshold) due to exposure to the disease and not vaccination
  - Definition of hospitalization; Hospitalized inpatients with (PCR) or without confirmed influenza; patients admitted to intensive care unit (ICU) or not; mechanically ventilated patients (“lung machines”) or not; inpatients vs outpatients
  - Definition of cause of mortality: Influenza and pneumonia (PI), excess mortality (PI, all causes of death etc.), respiratory diseases, pneumonia etc.
4. Baseline outcomes (control type), i.e. what was the control group or baseline outcome comparison? (general healthy population, infected patients, the hospitalized, patients with lab-confirmed seasonal influenza)
5. Independent variables of interest–relating to SES
  - Type of SES indices (education, income, crowding, density, deprivation index, unemployment, occupational social class, poverty status, % below poverty level)
  - Definition or brief descriptive text on SES indices (e.g., if based on a specific type of poverty index etc.)
6. Statistical methodology
  - Design of study (cross sectional, longitudinal, case-control, cohort studies)
  - Estimation technique (Cross tables, correlation analysis, OLS, Poisson regression, Logistic regression, Cox regressions, GEE regressions, GLMM models etc.)
  - Control variables included (e.g. age, gender, marital status, pre-existing disease, health behavior etc.) in light of sample restrictions (e.g. for pregnant women, sex is not among the controls)
  - Reference categories with which all point estimates are compared
7. Results reported (separate spreadsheet)

#### Data synthesis

Our narrative review includes a table of the study characteristics of the included studies, such as study authors and year, pandemic years, study region (region/country/hospital), sample inclusion criteria, sample size, unit of outcomes, data aggregation level, data sources and type, outcomes, baseline outcomes, SES measure, design, statistical techniques, controls, whether the study estimates are used in the meta analysis, and whether SES is an independent predictor. The quantitative part of the study pools results across individual studies using meta-analytic methods.

In the simplest meta-analytic model (“fixed effect”) random sampling variation is assumed to be the only source of variation in estimates. This is implausible in our context, in which studies use different SES indicators and medical outcomes from different countries and time periods. A “random effect” model captures the resulting *effect heterogeneity* by estimating the distribution of these underlying associations. Systematic variation across study-level covariates (e.g., pandemic period, region, type of outcome) is assessed using sub-sample analyses as well as a Bayesian hierarchical model.

We searched the identified studies in our meta-review for quantitative estimates of associations between SES indicators and influenza related outcomes. The resulting estimates were assessed for inclusion in the meta-analysis, and included if they could be expressed as an odds-ratio or relative risk for low versus high socioeconomic status. This implied that estimates had to include an indicator of socioeconomic status at the individual or ecological level, and had to allow for an estimate of how the incidence or prevalence of some flu related outcome varied by levels of this indicator. Where studies included estimates for distinct data subsamples (different age groups, periods), single estimates pooling all data were preferred if available. If not, the separate estimates were all included. For some studies, multiple estimates were also extracted if they performed different comparisons (e.g., risk of infection, and risk of hospitalization given infection). We also collected study level factors indicating the pandemic period (1918 vs 2009), country/region, and data as to whether the study estimate involved an odds ratio or a relative risk or rate. The specific studies included and all judgments and adjustments concerning inclusion and adjustments of reported numbers are detailed in the S2 File.

We have been inspired by NOS [21] to assess the quality of the included studies. We have rated the following items: A) *Selection* of exposed population (Broad and representative sample/population of the exposed?, truly and somewhat = 1, selected sub-groups and no description of population = 0) and non-exposed populations (1 = drawn from same community as the exposed, 0 = drawn from different source or no description); B) *Comparability* (confounder controls (yes = 1, no = 0), biological controls (yes = 1, no = 0), SES measure significant beyond biological controls (yes = 1, no = 0); C) *Data quality* (Lab-confirmed outcomes (yes = 1, no = 0) and data aggregation level (individual = 1, aggregate = 0)); The average and median quality score in the 44 studies included in the narrative review is respectively 4.5 and 4, while min score is 2 and max 7. The quality scores were higher in the 35 studies included in the meta-analysis (average 4.9, median 5, min 3, max 7) than those 9 not included (average 3.1, median 3, min 2, max 4) (see the quality assessment scores per article in the S2 Table).

Relative to the pre-analysis plan, the ambitions of the quantitative analysis were scaled back given the large heterogeneity across the studies included (see Table 1). The pre-analysis plan specified three types of analysis [22]. The first, a standard random effect analysis with subsample analyses, was conducted as planned using the «REML» algorithm in the Metafor meta-analytic package for R [23]. The second, a PET-PEESE analysis testing and adjusting for publication bias, was found unsuitable given the large effect heterogeneity [24]. The third, a Bayesian model to assess “dose-response” effects and assess how estimates vary with study-level indicators and the type of comparisons made, is included in a simplified version without the “dose-response” element.

**Table 1:** Overview of 44 studies included in the systematic review by study characteristics.

*	Study authors and year	Study region	Pandemic period	Sample inclusion criteria	Sample size	Unit of outcomes	Data aggregation level	Data sources	Outcomes	Baseline outcomes	SES measures	Design	Statistical technique	Controls	Estimates used in meta analysis and is SES an independent predictor?
2	[32]	London, England	20 April- 28 June 2009	People of all ages seeing a doctor for influenza at hospitals and community clinics in London	2,819 H1N1 patients (confirmed, presumed and probable) with valid LSOA postcodes	Individuals	Individual cases, but SES of cases based on the IMD of area post-codes	Data on cases and contacts were from the London Flu Response Center database and where coupled to IMD 2007	Influenza cases per 100,000	Population at risk in each LSOA area	Area Index of multiple Deprivation (IMD) 2007 quintiles (economic, social and housing issues)	Cross-sectional univariate design	Bivariate rate ratios with 95% CI	Age and weekly interactions with IMD	Meta analysis: Yes (all ages and whole period) SES measure significant
3	[33]	New York, USA	24 April-7 July 2009	Active hospitalized-based surveillance and passive collection of on demographics, risk conditions, and clinical severity	996 H1N1 patients (929 Confirmed and 67 probable)	Individuals	Individual cases, but SES of cases based on United Hospital Fund Poverty neighborhoods	Active hospitalized-based surveillance and passive collection of on demographic, risk conditions, and clinical severity	Hospitalizations per 100,000	Population at risk in high, medium or low poverty areas	Tertiles of percentage of residents living <200% of the federal poverty level according to the 2000 US Census	Cross-sectional univariate design	Bivariate Rate ratios with 95% CI	Age	Meta analysis: Yes SES measure significant
4	[34]	New Zealand	Nov 2009- March 2010	Randomly selected serum samples from GPs countrywide and in the Auckland region 3 months after the pandemic	1,687 serum samples	Individuals	Individual observations	seroprevalence data coupled with questionnaires evaluating demographics and potential risk factors.	H1N1 Infection rates (Seroprevalence; Antibody titer >1:40)	Baseline immunity was measured from 521 sera collected during 2004 to April-2009	Damp housing (poor housing conditions is an often used measure of SES, see [67])	Multi-stage random cross-sectional design	Multivariate logistic regressions	Age, ethnicity, gender, vaccination history, chronic illness	Meta analysis: Yes SES measure ns.
6	[35]	Eight cities in Hamedan Province, western Iran	July-December 2009	Subjects (cases and controls) were selected from patients with signs and symptoms of respiratory tract infection who were referred to health centers	245 cases and 388 controls	Individuals	Individual observations	Data are from health centers on H1N1 infection status coupled with covariate data from interviewers using predetermined questionnaires	Cases were identified by pharyngeal soap specimens positive for influenza A virus using PCR	Controls were testing negative for influenza A virus using PCR	Education 1. low education: illiterate, primary school and middle school. 2. High education: high school and academic	Unmatched case-control study	Multivariate logistic regressions	Age, sex, pregnancy, suspected close contact with influenza patients, smoking, region (urban rural), trip during last week, chronic disease, influenza vaccination, and BMI	Meta analysis: Yes SES measure significant, but unexpectedly higher risk for the high education group.
7	[26]	England & Wales	12 Oct 1918–5 April 1919	Influenza deaths in all parts of E & W	-	Aggregate: 305 adm. units & 62 counties	Aggregate	Deaths from National vital registration systems and demographic data from the 1921 census	Influenza death rates and reproduction number R (the average number of secondary cases generated by an index case)	Population at risk	People per acres, dwellings and rooms	Cross- Cross-sectional control-variable design	Spearman correlations, using a Bonferroni correction for multiple comparisons (transmissibility and death rates) and multivariate logistic regressions (death rates)	Population size, fall and winter waves, urban-rural	Meta analysis: No There were no association between transmissibility, death rates and indicators of population density and residential crowding
10	[36]	Global (226 studies from 50 countries met the inclusion criteria)	2009–2010	Described confirmed, probable or suspected cases of 2009–2010 influenza A (H1N1) infection; and (2) described patient(s) who were critically ill	10695	Individuals	Aggregate, Global	Medline, Embase, LiLACs and African Index Medicus to June 2009-March 2016	Mortality associated with H1N1-related critical illness	Population at risk	World Bank economic development status of countries (High, upper middle, lower middle income)	Systematic review and meta analysis	Random effects meta regressions	No controls	Meta analysis: No SES measure significant
11	[37]	Mexico	10 April to 13 July 2009	Data from clinical files from all influenza A deaths	239 H1N1 cases and 85 influenza A controls	Individuals	Individual observations	Patients’ clinical records and reporting forms from health facilities	Lab-confirmed A/H1N1 deaths (rt-PCR-test)	Seasonal influenza A deaths	Education (Primary school or less, Junior high school, High school or higher level)	Case-control	Propensity score multivariate logistic regressions	Sex, age, have a partner, smoking, employment status	Meta analysis: Yes SES measure ns.
12	[38]	Canada (Quebec)	16 April-1 July 2009	Lab-confirmed H1N1 hospitalizations or ICU admission/ deaths	321 hospitalized incl. 47 ICU and 15 deaths (cases) and 395 non-hospitalized N1H1 infection patients (controls)	Individuals	Individual observations	Suspected H1N1 case at primary care clinics or hospital coupled with other data from standardized questionaries’	Lab-confirmed influenza associated hospitalizations (24 hrs or more) and ICU/death	Non-hospitalized H1N1 patients (vs. hospitalized) or hospitalized non-severe (vs. ICU/death)	Education (high school not competed, non-University certificate, university degree)	Case-control	Multivariate logistic regressions	Age, sex, HCW, smoking, flu jab in 2008–09, consultation, days after onset, antiviral use, pregnancy, underlying condition, obesity	Meta analysis: Yes (both outcomes included) SES measure ns.
13	[39]	Spain (Andalusia, Basque Country, Catalonia, Castile and Leon, Madrid, Navarra and Valencia)	July 2009-Febr. 2010	Lab-confirmed hospitalization (RT-PCR)	699 hospitalized and 703 non-hospitalized cases of a(H1N1) infection	Individuals	Individual observations	Data from 36 hospitals and primary care centers in 7 spanish regions	Lab-confirmed hospitalizations (patient admitted to hospital for > 24 hours with RT-PCR confirmed H1N1 infection)	Non-hospitalized people with RT-PCR confirmed infection with the same pandemic virus	Education Secondary or higher	Case-control	Multivariate logistic regressions	Age, sex, ethnic group	Meta analysis: Yes SES measure significant. However, data on underlying health collected but not controlled for
14	[27]	USA (Chicago)	29 Sep-16 Nov 1918	Influenza and pneumonia (PI) mortality	7971 PI deaths	Individuals	Individual deaths, but SES measured at the level of 496 Census tracts	Historical maps of point-level mortality incidence, spatial data and near contemporaneous census data	Influenza and pneumonia mortality and reproduction number (R0)	Population at risk	Census tract-based SES (% illiteracy, unemployment, homeownership, population density)	Cross-sectional control-variable design	Poisson regressions with GEE and Spearman correlations	Age	Meta analysis: Yes % illiterate sig. predictor of mortality controlling for age and all other SES variables. Sig. ass btw. R0 and population density, illiteracy, and unemployment but not homeownership.
15	[40]	USA (Alaska, Arizona, New Mexico, Oklahoma, Wyoming)	15 April 2009–31 Jan 2010	Lab-confirmed A (H1N1) fatalities; state residents who died relating to infection with lab-confirmed influenza A	145 fatal cases and 236 controls	Individuals	Individual observations	Medical records (notifiable disease reports), death certificates, interviews with cases and controls	Lab-confirmed A(H1N1) fatalities using RT-PCR test	Outpatients with lab-confirmed H1N1	Healthcare insurance, >1,5 persons per room, graduated high school, poverty (<US$ 25000/year)	Matched case-control	Logistic regressions	Age, sex, race, barriers to health care access, urban-rural, health seeking behavior, vaccination status, health behaviors, pre-existing conditions.	Meta analysis: Yes (poverty) None of the SES variables were significant.
16	[41]	USA (23 counties)	23 April-8 June 2009	English language media reports of A (H1N1) cases	32 public primary & secondary schools with at least one confirmed H1N1 case and 6815 control schools located in the same 23 counties as the case schools	Aggregate, Schools	Aggregate	Health Map	Media reports of A (H1N1) cases	Schools located in the same 23 counties as the case schools without N1N1 cases	Title 1 school (Whether or not schools qualifies for a federal funding to support economically disadvantaged students.	Matched case-control	Logistic regression	Highest grade at school and size	Meta analysis: Yes SES measure significant
17	[42]	Australia (Brisbane)	Jan-Dec 2009	Lab-confirmed daily A (H1N1) cases	11,979 cases	Individuals	Individual cases, but SES measured for postcode areas (SLA)	Queensland Health, SEIFA data from Australian Bureau of Statistics (ABS) & daily rainfall & temperature data from the Australian Bureau of Meteorology	Lab-confirmed daily A (H1N1) cases	Rest of the population with no lab-confirmed case	SEIFA: socioeconomic index for areas, incl. education, occupation and wealth	Cross-sectional control-variable design	Bayesian spatial conditional autoregressive poisson models	Rainfall (mm) and temperature (degrees Celsius)	Meta analysis: No SES measure ns.
18	[43]	Australia (Queensland)	7 May-31 Dec 2009	Lab-confirmed A (H1N1) cases	-	Individuals	Individual cases, but SES measured for postcode areas (SLA)	Queensland Health, SEIFA data from Australian Bureau of Statistics (ABS) & daily rainfall & temperature data from the Australian Bureau of Meteorology	Lab-confirmed daily A (H1N1) cases	Rest of the population with no lab-confirmed case	SEIFA: socioeconomic index for areas, incl. education, occupation and wealth	Cross-sectional control-variable design	Flexible Bayesian, space-time. SIR models	Rainfall (mm) and temperature (degrees Celsius)	Meta analysis: No SES measure significant
19	[44]	England (West Midlands)	16 April-6 July 2009	Lab-confirmed A (H1N1) cases	3063 cases	Individuals	Individual cases, but SES measured for postcode areas	FluZone, a national surveillance database with case reporting. SES data from IMD 2007	Lab-confirmed A (H1N1) cases	Rest of the population with no lab-confirmed case	Index of Multiple Deprivation of an area and postcodes (IMD 2007). It includes seven dimensions: income, employment, health deprivation and disability, skills and training, barriers to housing and services, crime and disorder, living environment SES indexes IMD 2007: Index of Multiple Deprivation	Cross-sectional	Descriptive analysis	Age, sex, ethnicity, exposure and illness severity, but no controls were made	Meta analysis: No SES measure significant
21	[45]	Canada (Rural community of British Columbia; local town and surrounding First Nation reserves	Late April/early May 2009	One elementary school and on-reserve aboriginal participants;	83 ILI cases and 281 non-ILI cases	Individuals	Individual observations	Phone survey of households with at least one child enrolled in any of the community schools	Influenza-like illness (ILI)	Non-ILI cases	Household density	Cross-sectional control-variable design	Generalised linear mixed models (GLMM)	Age, chronic conditions, aboriginal status, received vaccination 2008–09	Meta analysis: Yes SES measure ns.
23	[46]	Spain (Andalusia, the Basque Country, Castile and Leon, Catalonia, Madrid, Navarre, and Valencia	July 2009- Feb. 2010	Patients aged 6 months to 18 years with confirmed H1N1 at 32 Hospitals of the Spanish National Health survey	195 confirmed H1N1 hospitalized cases and 184 outpatient controls with confirmed H1N1	Individuals	Individual observations	Spanish National Health Service	Lab-confirmed A (H1N1) inpatient (hospitalized) cases	Outpatient (non-hospitalized) controls with confirmed H1N1	Parents education (Primary or lower vs. secondary or higher)	Matched case control, prospective, observational study	Logistic regressions	Age, pulmonary, disease, neurological disease, diabetes mellitus, cardiovascular disease, and non-Caucasian ethnicity	Meta analysis: Yes SES measure significant
24	[47]	Brazil (Paraná)	2009	Patients (in- and outpatients) with lab-confirmed H1N1 infection	1911 Inpatient cases and 2829 outpatients controlls	Individuals	Individual observations	Brazilian Ministry of Health National Case Registry Database	Lab-confirmed A (H1N1) inpatient cases and outpatient controlls	Lab-confirmed H1N1 outpatients controlls	Level of education (Literate vs. illiterate)	Retrospective observational case-control study	Logistic regressions	age, gender, ethnicity, having a comorbiditiy, number of comorbiditis, 8 types of underlying health conditions, smoking, clinical manifestations, treatment (Oseltamivir), time to treatment initiation in days	Meta analysis: Yes SES measure significant
26	[48]	USA (New York)	1 Oct 2009–28 Feb 2010	Lab-confirmed illness among adults and children	128 inpatients with lab-confirmed flu cases matched by age and month of diagnosis with 246 non-hospitalized lab-confirmed influenza A controls (assumed to be H1N1)	Individuals	Individual observations	Sentinel surveillance system used by NYC Department of Health and Mental Hygiene; telephone interview to collect clinical and demographic data	Lab-confirmed A (H1N1) inpatient cases and outpatient influenza A controls	Non-hospitalized lab-confirmed influenza A controls (assumed to be H1N1)	Education (Some college or more, not a high school graduate, high school graduate), annual household income and neighbourhood poverty (% Persons living below the federal poverty line)	1:2 case-control study design, matching by age group and month of diagnosis	Conditional multivariate logistic regressions	Access to care (primary physician, insurance) and at least one underlying condition (various diseases, pregnancy and obesity)	Meta analysis: Yes Education among adults and neighbourhood poverty among children and adults were significant
28	[49]	Canada (Ontario)	Two waves in 2009 (April 23-July 20 and August 1 Nov 6)	Residents of all ages who received nasopharyngeal swabs and tested positive for H1N1	401 self-reported hospitalization cases and 624 non-hospitalized controls (150 hospitalized and 184 non-hospitalized in wave 1, 251 hospitalized and 440 non-hospitalized in wave 2)	Individuals	Individual hospitalizations by individual-level education and contextual level SES variables	Surveillance data and standardised phone interviews	Lab-confirmed A (H1N1) inpatients (hospitalized patients)	Non-hospitalized controls H1N1 positives	individual level education level (of adult participants aged 18 years or older & of parents respondents for children younger than 16 years), household density (individuals per sleeping rooms) and several contextual level SES variables (employment, education, income, social and material deprivation)	Case-control study	Binomial or multinomial logistic regression, using generalized estimating equations to account for clustering/dependence in the data	Age and gender	Meta analysis: Yes (Total deprivation and individual and parental education for both waves). First wave: High school education or less and living in a neighborhood with high material or total deprivation sign. Second wave: High school education or less sign. Moreover, a mediation analysis showed that clinical risk factors explain only a portion of the ass. btw SES & hospitalization.
29	[50]	USA (California)	3 April-15 Sep 2009	Reported counts of H1N1 hospitalizations, not lab-confirmed	2010 hospitalizations	58 counties	Aggregate	California Department of Public Health surveillance data	Reported H1N1 Hospitalizations	Population at risk in each 58 counties	Education (% of persons aged > 25 years with a high school diploma); Poverty (% of pop under poverty line); Income (median HH income in dollars)	Cross-sectional control-variable design	OLS	Sex, race/ethnicity, age, climate, agricultural and transportation variables	Meta analysis: No The 3 SES variables were ns. but results not shown
30	[12]	Norway	1918–1919	PI deaths covering the whole of Norway	16,005 deaths	Aggregate, 351 medical districts	Aggregate	Regional district physician reports and census data	PI mortality reported to a doctor	Population at risk	% receiving public support due to poverty; Wealth per person (in 1000 Nok); Average number of persons per room	Cross-sectional control-variable design	OLS	age, sex, ethnicity, % in fishing, coast-inland, summer wave exposure	Meta analysis: No Poverty and wealth, but not crowding was sign.
31	[13]	Norway (Frogner and Grønland/Wexels parishes in Oslo)	1 Feb 1918–1 feb 1919	PI deaths in the two selected parishes	250 PI deaths	Individuals	Individuals	Death certificates coupled with census data	PI mortality reported on death certificates	Population at risk	Occupational based social class, apartment size (1–8 rooms +) and parish	Longitudinal multivariate survival analysis	Cox regressions	Age, sex, marital status	Meta analysis: Yes (occupation based social class) Apartment size and parish but not occupation-based social class was sign.
34	[51]	Spain (Andalusia, the Basque Country, Castile and Leon, Catalonia, Madrid, Navarre, and Valencia)	July 2009-Feb 2010	Patients recruited from hospitals & primary health care clinics & emergency units during the peak of the influenza A 2009 pandemic in	699 hospitalized and 699 non-hospitalized with Lab-confirmed cases A(H1N1) cases using (RT-PCR)	Individuals	Individuals	Cases filled in a questionaries’ at the health centre or by phone to obtain covariate information	Hospitalized lab-confirmed A (H1N1) cases	Non-hospitalized (family physician visits at primary health care clinics and emergency units) cases of A(H1N1) infection	Education (Secondary or higher vs no formal education or primary education) and overcrowding (below the fifth percentile of the distribution of square metres available per person in the normal residence of all study participants)	Multicenter Matched case-control (according to age, date of hospitalisation in of the case (+/- 21 days) & province of the residence of the case)	Binomial logistic regression using Cox conditional logistic regressions	Sex, ethnicity, prior preventive information, prior pandemic vaccination, previous outpatient care or emergency care and unfavourable medical factors (smoking, morbid obesity (BMI >40), hypertension, lung disease, cardiovascular disease, kidney failure, diabetes, chronic liver disease, immunodeficiency, disabling neurological disease, malignancy, transplantation, cognitive dysfunction, seizure disorders and rheumatic diseases)	Meta analysis: Yes (education) Education decreases & Overcrowding increases outcome significantly
35	[14]	Global study covering 27 countries with high-quality vital registration data for the 1918–1920 pandemic	1918–20	Data for populations where vital registrations are believed to be more than 80% complete, supplemented with subnational data for US states & provinces of "pre-partition" India	27 countries for 1918–1920, 24 US states with data available for the period, and nine Indian provinces	Countries and states	Aggregate	Human mortality database, B.R. Mitchels International Historical Statistics Series, subnational data from US states and provinces of prepartition India	Excess mortality by comparison of annual death rates during the pandemic to the average of annual death rates before and after the pandemic	Population at risk	Income (Per-head income in real international dollars (corrected for price changes)	Cross-sectional control-variable design	OLS with log of pandemic mortality and log income and absolute value of latitude	Latitude, to control for diurnal temperature fluctuation	Meta analysis: Yes Log per-head income in 1918 sign.
36	[52]	Canada (Ontario)	13 April-20 July 2009	Residents (children and adults) tested for A(H1N1) using RT-PCR	240 cases and 112 controls among children (< 18 years) and 173 cases and 229 controls among adults (>18 years)	Individuals	Individuals H1N1 status by individual education and several ecological SES variables	Clinic-based sample from Ontario, individuals presented to clinics for medical care + standardised telephone interviews	Lab-confirmed 2009 pandemic cases	RT-PCR negative H1N1 cases	Individual Education (high school or less and post-secondary school completion) Area measures: Material, social, total, low employment rate, low income.	Test-negative case-control study	Logistic regressions	age, gender, bmi, ethnicity, current smoker, underlying medical conditions, household density, children in household, receipt of 2008 seasonal vaccine, tested prior to 11th June 2009, healthcare provider, Toronto residence, immigrant category	Meta analysis: Yes (Total deprivation, one for adults and one for children). None of the SES variables were sign. in univariate models and were therefore not entered in the multivariate models.
37	[53]	Europa (30 EU/EFTA countries)	May 2009-May 2010	Confirmed and notified fatal pandemic influenza A(H1N1) deaths in EU/EFTA region	2896 fatal cases	Aggregate, Countries	Aggregate	ECDC and Eurostat	Lab- confirmed and notified deaths	Population at risk	GDP per capita	Cross-sectional control-variable design	Random effect Poisson regressions	greenhouse gas emissions, concertation of particular matter, latitude, hospital beds per 100,000 inhabitants, per capita government expenditure on health, unmet need for medical examination/treatment, Gini coefficient, employment rate, proportion of population aged 65+, old age dependency ratio, women per 100 men	Meta analysis: Yes GDP per capita was sign. in univariate model, but not in multivariate model.
38	[54]	Australia (Barwon statistical division in Southeastern Australia)	Sep 2009-May 2010	Adult subjects in Geelong Osteoporosis Study, a group randomly selected from electoral rolls, were invited to participate in this sub-study to provide blood samples and complete a questionnaire. Sample of seropositive adults prior to the availability of a vaccine	1184 individuals (129 seropositives and 1055 seronegatives)	Individuals	Individual seropostive status by ecological SES variables	Blood samples and self-report questionnaire	Haem agglutination inhibition test, seroposotivity was defined as a titre > 1:40	Seronegative persons	Australian Bureau of Statistics’ Index of Relative Socioeconomic Advantage and Disadvantage (IRSAD) Area-level measure of education, occupation, income, unemployment and household structure (quintiles 1–5)	Cross-sectional control-variable design	Multivariate logistic regressions	age, bmi, obese, current smoker, healthcare worker, childcare worker/teacher, employment status, highest level of education, lives alone, lives with children aged <12 years, chronic respiratory disease, pregnancy, chronic heart disease, diabetes	Meta analysis: Yes The SES variable was significant in multivariate models
39	[31]	England and Wales (62 of 82 counties)	Week ending 29 June 1918 to 10 May 1919	Counties with SES info from 2000 which could be linked to counties in 1918	Sample covers 333 units and 62 out of 82 counties	Individual deaths	Aggregate	Weekly influenza deaths & annualised rates/1000 population, collated by the Registrar General’s Office in 1920	Influenza mortality	Population at risk	The average of Ward Scores from the Indices of Deprivation 2000: District level Presentations for England It combines a number of indicators which cover a range of domains (Income, Employment, Health Deprivation and Disability, Education, Skills and Training, Housing and Geographical Access to Services) into a single deprivation score for each area.	Cross-sectional control-variable design	non-parametric Spearman correlation coefficient	Pre-pandemic mortality, age, population size (persons/acre)	Meta analysis: No SES measure sign. in waves 1 and 3, but not wave 2
40	[55]	USA (state of Massachusetts)	26 April-30 Sep 2009 (before the vaccine became available)	Patients met the following inclusion criteria: 1) Patients were discharged from acute care hospital. 2) assigned 1 or more diagnosis codes corresponding to a grouping of ICD-9. 3) younger than 65 years	4874 hospitalizations of which 526 admitted to ICU	Individuals	Individual hospitalizations, but area-level SES variables	Linked hospital discharge and American Community Survey and US Census data	Lab-confirmed H1N1 ICU stays	Hospitalized non-ICU patients	% of pop below poverty level 2006–2010 for zip code areas	Cross-sectional control-variable design	Logistic regressions	Racial/ethnic groups, gender, age, admission though EP/OP	Meta analysis: Yes Unexpectedly, those in less affluent SES groups had sign. lower risk of ICU stay than the most affluent SES group
41	[56]	USA (341 US counties in 14 states)	July 2009-June 2010	Only states with consistent reporting and updating of H1N1 statistics, that is reporting standards met by the CDC	Sample size not given.	Aggregate,341 counties	Aggregate	County-level H1N1 deaths are from CDC and SES variables from US census and CDC 11% of US counties covered, SES measures are representative to similar characteristics to USA as a whole	H1N1 deaths according to CDC	Population at risk	Per capita personal income; median household income; educational attainment (persons aged >/ = to 25 years), percent high school graduate or higher, educational attainment (persons aged >/ = 25 years), percent bachelor’s degree or higher; people of all ages in poverty (%)	Univariate and cross-sectional design	Correlations	No controls	Meta analysis: Yes In univariate models poverty positively predicted mortality while income and education variables negatively predicted mortality. Multivariate modelling was not carried out.
42	[57]	Spain (Andalusia, the Basque Country, Castile and Leon, Catalonia, Madrid, Navarre, and Valencia)	July 2009-Feb 2011	Cases and controls were aged > 18 years and picked from 36 hospitals and 22 primary-care centres	715 primary care centre H1N1 cases, 715 other diseases than ILI primary centre controls, and 406 hospitalized H1N1 cases	Individuals	Individuals	Hospital and primary care data	Lab-confirmed H1N1 cases and hospitalizations (RT-PCR)	Infection model: Controls were primary care patients with other disease than ILI Hospitalization model: cases were primary care centre H1N1 cases	Occupational based social class (Manual vs. non-manual workers)	Matched case-control study	Logistic regressions	In model for infection: age, pregnancy, diabetes and influenza vaccination. In hospitalization model: age, pregnancy, COPD, cardiovascular disease, diabetes, and influenza vaccination	Meta analysis: Yes SES variable sign. in multivariate models for both infection and hospitalization risks
44	[17]	England	1 June 2009–18 April 2010	All deaths reported due to pandemic flu	349 out of 365 deaths (95,6%) in England	Individual deaths	Aggregate: Individuals aggregated up to five approximately equal population groups to create area deprivation quintiles	National Health Service; basic set of demographic information	Pandemic deaths, no info whether these were lab-confirmed or not, but they were probably lab-confirmed	Population at risk	Index of Multiple Deprivation of an area and postcodes (pooled measure based on income, education, housing, health and crime) (1–5, where 5 is least deprived and 1 most deprived)	Cross-sectional table analysis	Direct age-sex standardization of mortality rates using mid-point 2009 pop estimates for England	Age, sex, and Urban and rural areas	Meta analysis: Yes SES variable significant with and without urban-rural interactions
45	[58]	Global: 20 countries covering 35% of the world population	2009 pandemic mortality	Weekly virology and underlying cause-of-death mortality time series for 2005–2009	123,000–203,000 deaths in the last 9 months of 2009	Aggregate	Aggregate	Weekly virology data from the WHO FluNet and national mortality time series	Excess mortality associated with the 2009 pandemic	Population at risk	Gross national income (GNI) per capita (US dollars	Univariate cross-sectional time-series analysis	Multivariate OLS regressions	-	Meta analysis: No. Coefficients not given in the paper or in online appendix Estimates between Gross national income and mortality was ns.
48	[28]	New Zealand	27 Aug 1918-March 1919	Male soldiers (New Zealand Expeditionary Forces (NZEF) in both hemispheres in 1918–1919 pandemic period)	930 deaths, taken from 1000 randomly selected records	Individuals	Individuals	Death certificates	Influenza, pneumonia, and bronchitis deaths	NZEF population at risk	Pre-enlistment occupational based social class (1–3 (most privileged), 4–6 and 7–9 (least privileged)	Univariate cross-sectional design	Univariate Rate ratios	No controls	Meta analysis: Yes SES measure not significant
49	[29]	New Zealand	20 July-13 Oct 1918	Male navy soldiers (military personnel in HM New Zealand Transport troop ship Tahiti)	77 deaths, 1117 military personnel plus 100 crew (total pop at risk 1217)	Individuals	Individuals	Death certificates	Influenza and pneumonia deaths	Population at risk at HM New Zealand Transport troop ship Tahiti	Occupation-based social class (1–6 and 7–9 (1 is company manager and 9 is labourer)	Cross-sectional control-variable design	Multivariate logistic regression	age, military rank, rurality score, military unit	Meta analysis: YES SES measure not significant
50	[30]	USA (New London, Connecticut, Baltimore, Maryland, Augusta, Georgia, Macon, Georgia., Des Moines, Iowa, Louisville, Kentucky, Little Rock, Arkansas, San Antonia, Texas, San Francisco, California	1 Sep-Dec 1918	Nine urban localities with a population of at least 25,000, randomly selected, only white populations	94,678 individuals, 26,824 morbidity cases (influenza, pneumonia and "doubtful" cases), X deaths	Individuals	Aggregate	Survey data (e.g. Baltimore: sample 33,776 (5.68% of pop)	Self-reported pandemic morbidity, mortality and case fatality rates (	Morbidity: Population at risk in canvassed areas and lethality: mortality among the sick	Economic status (Very poor; poor; moderate; well-to-do (based on the enumerators impression)	Cross-sectional control-variable design	Cross-tables and direct standardization techniques to control for age-differences etc.	age, sex, size of household	Meta analysis: Yes SES measure sign. related with both outcomes.
51	[16]	USA (New Haven County, Connecticut)	2009–10	Hospitalized, laboratory confirmed influenza among adults 18 years and older	213 hospitalizations	Individuals	Individual lab-confirmed hospitalizations but neighbourhood level SES measures (185 Census tracts)	Surveillance data (Connecticut Emerging Infections Program’s influenza-associated hospitalisation surveillance system) + chart reviews & interviews with healthcare providers & with patients or their proxies. Census tract level data obtained from the US Census Bureau’s 2006–2010 American Community Survey (ACS)	H1N1 lab-based hospitalizations	Population at risk in New Haven	Below federal poverty, no high school diploma, median income	Cross-sectional design	Age-adjusted incidence of influenza-associated hospitalizations among adults by neighbourhood SES characteristics.	Age.	Meta analysis: Yes All 3 SES measures are sign. and display a clear social gradient
52	[59]	USA (state of New Mexico)	14 Sep 2009–13 Jan 2010	Hospitalized, positive influenza hospitalization, Mechanical ventilation and death among the hospitalized	926 lab-confirmed H1N1 hosp. Patients, 106 mechanically ventilated and 35 deaths	Individuals	Individuals outcomes, but 33 counties divided into 4 quartiles by median household income	New Mexico Department of Health statewide surveillance of hospitalizations and deaths. Estimates from the US Census Bureau’s Small Area Income and Poverty Estimates programme.	H1N1 related hospitalisations, mechanical ventilation and death	Comparison group for hospitalizations: general statewide population; Comparison group for mechanical ventilation and death among those hospitalized were the hospitalized	Household Income (County median household annual income quartile)	Cross-sectional control-variable design	Poisson and logistic regressions	Hospitalization model: age, gender, and race/ethnicity. Mechanical ventilation model: age, gender, and race/ethnicity, obesity, high risk conditions, neuraminidase treatment, time from illness onset to seeking medical care. Mortality risk model: ns in unadjusted model, therefore no multivariate model	Meta analysis: Yes SES measure sign. in model for hospitalization risk but not in models for mechanical ventilation and death
53	[60]	Canada (Winnipeg, Manitoba)	Oct- Dec 2009	Adults presenting to three inner city community clinics were recruited as study participants using convenience sampling.	458 study participants (174 participantsOct-12 Nov, before the vaccine was available), 206 cases 13 Nov-Dec, which did not get take the vaccine; 78 participants enrolled on or after Nov 13 which did get the vaccine are not included in our meta-analysis)	Individuals	Individuals	Serological testing and questionnaire data	Seropositive cases	convenience sample population at risk	Education (High school or not) and annual household income	Univariate & cross-sectional analysis	Prevalence estimates with exact binomial 95% CI using Clopper Pearson intervals	no controls	Meta analysis: Yes The two SES measures ns. for both periods.
55	[61]	Australia (Northern Territory)	2009 (June-August)	Antibody titers were determined by hemagglutination inhibition against reference virus A/California/7/2009 on serum samples collected opportunistically from outpatients	1689 serologic specimen post pandemic (cases 3–30 September 2009) and 445 serological specimen prepandemic (controls January 10 to May 29, 2009)	Individuals	Individual seropositive status but SES measure is aggregate	Serological data, specimens from pathology lab, and computer matching of data to indigenous status and SEIFA measures	lab-confirmed seropositivesand attack rates (difference between post and pre-pandemic immunity)	serological specimen prepandemic (controls January 10 to May 29, 2009)	2006 Statistical Local area (SLA) was linked to Australian Bureau of Statistics’ Socio-Economic Indexes for Ara (SEIFA). SEIFA measures (quintiles) use information from census data relating to material and social resources and ability to participate in society to obtain a broad level of relative socioeconomic status for each SLA	Case-control design	Logistic regressions	age, gender, aboriginal and Torres strait islanders, region	Meta analysis: Yes SES measure ns.
57	[62]	Canada (province of Manitoba)	2 April-5 Sep 2009	Confirmed H1N1 cases for whom the final location of treatment was known	795,569 community cases, 181 hospitalized but not ICU, 45 admitted to ICU	Individuals	Individual H1N1 case status, but area income quintiles	Lab-confirmed H1N1 data, hospital data and data collection–form completion via interviews	lab-confirmed community cases, hospitaliations and ICU admissions	Two control groups. Community cases (vs. hospitalizations) and hospitalized, non ICU (vs. ICU).	Income based on postal codes (Top three quintiles vs the bottom two quintiles)	Cumulative case-control design	Logistic regressions	Age, gender, pregnancy, ethnicity, any comorbidity, Interval from symptom onset to antiviral treatment, rural vs urban	Meta analysis: Yes SES measure ns. in models for both hospitalizations and ICU admissions
58	[63]	China (Beijing)	1 Aug-30 Sep 2009	Households of hospital healthcare workers. Case households were: (1) has an index patient of H1N1. (2) index case was quarantined in household from onset of diagnosis to 7 days after onset of illness; (3) secondary case had potential contact with index patient; (4) symptoms onset of secondary case occurred within 7 days since last known contact with index case during infectious period of index case; (5) RT-PCT confirmation date of secondary case occurred within 7 days since last known contact with index case during infectious period of index case; (6) none of the household members previously received a vaccine against pandemic H1N1 2009 influenza	54 case households (HH with a self-quarantined index patient and a secondary case), 108 control households (HH with a self-quarantined index patient and a close contact)	Individuals	Households	Household transmission data	Lab-confirmed secondary cases (RT-PCT)	Households with a self-quarantined index patient and a close contact	Education (High school and higher vs middle school and lower)	1:2 matched case-control design	Conditional logistic regression	Sharing room with index case-patient; Ventilating room every day; and Frequency of hand washing	Meta analysis: Yes SES measure significant
59	[64]	England	27–30 April 2009	Lab-confirmed AH1N1 pandemic flu deaths	337 of 389 lab-confirmed fatalities (86.6%)	Individuals	Individual lab-confirmed deaths, but SES is measured for 32378 super output areas (LSOA)	National Health Service	lab-confirmed deaths	Population at risk	Index of Multiple Deprivation of an area and postcodes (IMD 2007). It includes seven dimensions: income, employment, health deprivation and disability, skills and training, barriers to housing and services, crime and disorder, living environment	Cross-sectional control-variable design	Poisson regressions	Age, gender, rural vs urban	Meta analysis: Yes SES measure significant.

* These numbers correspond to the 59 studies from which we extracted data. In the data extraction phase, we removed an additional 15 studies The final number of studies included in the narrative synthesis was therefore the 44 listed in this table, also see documentation in S2 File.

The Bayesian model differs from the standard fixed and random effect models in two ways. It allows us to include study-level covariates to capture systematically different effects in specific regions, periods or for specific outcomes, using a hierarchical specification across the parameters to impose partial pooling and reduce the risk of large but spurious estimates [25]. If the evidence as a whole indicates that estimates vary no more across study level indicators than we would expect due to sampling variation, then this will pull the individual indicator coefficients towards zero.

Second, the Bayesian model requires a prior distribution for each model parameter that expresses reasonable (pre-analysis) beliefs regarding the parameter values. The estimation updates these beliefs in light of the data, resulting in a posterior distribution that blends the pre-existing knowledge encoded in the prior distribution with evidence from the observed data. To verify that the prior choices for the overall pooled effect and heterogeneity do not unduly influence the result, the Bayesian model is also estimated without study-level covariates to allow for comparison with the standard random effect model.

## Results

### Narrative review

#### Flow of included studies

Our database search identified 8,411 records. After leaving out duplicates, 4,203 studies were imported for screening. After removing another 75 duplicates, we screened the titles/abstracts of 4,128 records. Of these, 3952 studies were irrelevant, and 176 full text studies were then assessed for eligibility. In this phase, 117 studies were excluded, leaving us with 59 studies from which to extract data. In the data extraction phase, we removed an additional 15 studies. The final number of studies included in the narrative synthesis was therefore 44 (see PRISMA Flow Chart in S1 File).

#### Study characteristics

The review identified a total of 44 studies, 9 studies of “Spanish flu of 1918–20” [12–14, 26–31] and 35 of the “Swine flu of 2009–2010” [15, 16, 32–64] (Table 1). We identified no studies of the Russian flu of 1889–90, the Asian flu of 1957–58 or Hong-Kong flu of 1968–70. Most of the studies used data from North America, including 11 for USA [16, 27, 30, 33, 40, 41, 48, 50, 55, 56, 59] and 6 for Canada [38, 45, 49, 52, 60, 62]; Europe, including 6 for England [15, 26, 31, 32, 44, 64], 4 for Spain [39, 46, 51, 57], 2 for Norway [12, 13], and 1 for 30 EU/EFTA countries [53]; 4 for Australia [42, 43, 54, 61] and 3 for New Zealand [28, 29, 34]. While a few studies used data from Central America/South America including 1 for Mexico [37] and 1 for Brazil [47], and Asia, including 1 for Iran [35] and 1 for China [63], we identified no studies using data from Africa. Finally, 3 studies had a global approach studying several countries [14, 36, 58].

The sample inclusion criteria varied greatly from study to study. Two of the 44 studies studied military populations, one of these studied mortality in randomly selected records [28], the other studied mortality on one transport troop ship [29]. Of the 42 studies using civilian study populations, some studied particular patient populations/cohorts [46, 54, 61, 63], general patients at various hospitals and health centres [16, 32, 33, 35, 39, 40, 47–49, 51, 52, 55, 57, 59, 60, 62], students at schools or students including their families [41, 45], or general populations living in various cities, states, counties or (several) countries [12–15, 26, 30, 31, 34, 36–38, 42–44, 50, 53, 56, 58, 64].

The sample size in each study varied substantially and is reported in Table 1 whenever information was available for the pandemic events (for cases and controls) and the population at risk.

The unit of the outcome variables was either individual in 36 studies [13, 15, 16, 27–40, 42–49, 51, 52, 54, 55, 57, 59–64] or aggregate in 8 studies [12, 14, 26, 41, 50, 53, 56, 58]. Some of the studies with individual-level outcome data nevertheless preformed analysis at aggregated levels. In 12 studies the data aggregation level was aggregate [12, 14, 15, 26, 30, 31, 36, 41, 50, 53, 56, 58]. 15 studies had individual-level outcome variables and control variables, but used area-level (and individual-level) SES variables [16, 27, 32, 33, 42–44, 49, 52, 54, 55, 59, 61, 62, 64]. Studies using only ecological SES variables thus picked up a combination of individual-level and area-level SES effects on the outcome variables. Finally, in 17 of the studies, outcomes, explanatory variables and controls were all measured for individuals and the data aggregation level was thus the individual level [13, 28, 29, 34, 35, 37–40, 45–48, 51, 57, 60, 63].

There were generally three types of data source used in the 44 studies included in the narrative synthesis: 1) 28 studies used active surveillance of events coupled with SES and covariate data via questionnaires, face-to-face or telephone interviews or censuses [16, 32–44, 46–52, 54, 57, 59–63]; 2) 14 studies used national vital registration systems on events coupled with SES and covariate data via censuses [12–15, 26–29, 31, 53, 55, 56, 58, 64]; 3) 2 studies used telephone survey or data collected via door-to-door survey to collect both event and population at risk data [30, 45].

The 3 broad categories of outcomes were studied (see details in Table 1): 1) people seeing doctors due to symptoms of influenza like illness (ILI)/influenza transmission(R0)/lab-confirmed influenza infection (using PCR tests)/immunity towards influenza (using blood serum samples to look for antibodies) [26, 27, 30, 32, 34, 35, 41–45, 52, 54, 57, 60, 61, 63]; 2) lab-confirmed influenza hospitalizations/ICU treatment/mechanical ventilation [16, 33, 38, 39, 46–51, 55, 57, 59, 62]; 3) lab-confirmed pandemic deaths/Influenza-Pneumonia (PI) deaths/excess deaths associated with pandemic influenza [12–15, 26–31, 36, 37, 40, 53, 56, 58, 59, 64].

The choice of baseline outcomes (or controls in case-control studies) partly depended on the outcomes studied, and included: 1) General population at risk [12–16, 26–33, 36, 50, 53, 56, 58–60, 64]; 2) General population at risk without H1N1 Infection or ILI [41–45]; 3) Patients with ILI, persons in quarantine for a suspected case and a close H1N1 contact or patients with ILI testing negative for influenza A H1N1 infection [30, 35, 52, 63]; 4) pre-pandemic immunity [34, 61]; 5) seasonal influenza A deaths [37]; 6) Non-hospitalized H1N1 positive patients or hospitalized H1N1 positive non-severe (not ICU or death) [38, 39, 55, 59, 62]; 7) Outpatients with H1N1 infection [40, 46–49, 51, 57]; 8) Seronegative for H1N1 [54]; 9) Patients with other diseases than ILI [57].

The studies that used individual-level SES measures used one or several of the following; (household) income [40, 48, 60], economic status [30], education [35, 37–40, 46–49, 51, 52, 60, 63], occupation-based social class [13, 28, 29, 57], size of apartments, poor housing or crowding measures [13, 26, 34, 40, 45, 49, 51], and having health insurance [40]. Some used both individual-level and area-level measures of SES. The SES measures used at the area-level were often (but not always) indexes of economic, social and housing deprivation/development [12, 14–16, 27, 31–33, 36, 41–44, 48–50, 52–56, 58, 59, 61, 62, 64].

The 44 studies included in the review used study designs that fall into four categories: 1) Systematic review and meta-analysis [36]; 2) Cross sectional univariate or control-variable design [12, 14–16, 26–34, 42–45, 50, 53–56, 59, 60, 64]; 2) Case-control design [35, 37–41, 46–49, 51, 52, 57, 61–63]; 3) Longitudinal survival analysis [13]; 4) Time-series analysis [58].

The identified studies were descriptive or explanatory. The descriptive studies used statistical techniques to calculate pandemic disease burden estimates and univariate correlations between the outcomes and various variables as well as demographic standardization techniques to control for age and sex [15, 16, 28, 30–33, 44, 56, 60]. The explanatory multivariate studies used modelling techniques such as OLS [12, 14, 50, 58], generalized linear mixed models [45], logistic regressions [26, 29, 34, 35, 38–41, 46–49, 52, 54, 55, 57, 59, 61–63], propensity score logistic regressions [37], Poisson regressions [27, 53, 59, 64], Cox regressions [13, 51], random effect meta-regressions [36], and various types of Bayesian models [42, 43].

#### Study results

The results in the 9 identified studies on the 1918 influenza and SES were mixed [12–14, 26–31]. After various controls were made, 6 studies found a significantly and expected *higher* mortality for *lower* SES groups [14] or higher mortality/transmission rates, but not a clear social gradient for all SES measures [12, 13, 27]; a significant *higher* mortality for *lower* SES groups, but only for 2 out of 3 pandemic waves [31]; or a significantly *higher* morbidity and mortality for *lower* SES groups [30], while 3 studies found no association between SES and mortality [28, 29] or mortality and transmission rates [26]. However, none of the 6 studies documenting significant associations with a higher pandemic disease burden for lower SES groups included data to control for medical risk factors. Hence, some or all of the identified associations between SES and the pandemic outcomes in the 6 above mentioned studies could potentially have been “explained away” by controlling for having latent tuberculosis [65] or other known comorbidities [66].

Fourteen of the 35 identified studies on the 2009 pandemic had data to adjust for both medical and social risk factors [34, 35, 38, 40, 45–48, 51, 52, 54, 57, 59, 62]. After adjusting for medical risk factors, 7 of these studies documented independent and *expected* impact of SES (*higher* risks for *lower* SES) on either infection/immunity [34, 54], hospitalization [46–48, 51] or both of these outcomes [57]; 1 study found both expected significant associations with SES (higher risk of hospitalization) and non-significant (ICU and death) impact of SES after medical risk factor were controlled for [59]; 5 studies found non-significant effects of SES on ILI/infection/immunity [45, 52], hospitalization/ICU [38, 62] and mortality [40]; and finally, 1 study found a significant but *unexpected* impact of SES on infection, that is *higher* infection rates for those with *high*er vs. lower education [35]. Although the findings in these 14 studies investigating both social and medical vulnerabilities were somewhat mixed, they show that medical risk factors are not simply 100% correlated with socioeconomic factors, and in 8 of these 14 studies social factors explained variation in the variation in the pandemic outcomes beyond that captured by medical factors.

21 of the 35 identified studies on the role of SES in the 2009 pandemic outcomes *did not* control for medical risk factors but found the following. First, 12 studies found significantly *higher* risks for the *lowest* socioeconomic status group, of which 5 studied ILI/infection/immunity [32, 43, 44, 63]; 4 investigated hospitalizations [16, 33, 39, 49]; and 4 studied mortality [15, 36, 56, 64]. Second, 7 studies found non-significant associations with SES, of which 2 studied ILI/infection/immunity [42, 60]; 2 studied hospitalizations [50, 62] or ICU treatment [62], and 3 studied mortality [37, 53, 58]. Finally, 2 studies unexpectedly found respectively a higher risk of a lab-confirmed case [41] or ICU treatment [55] in the *highest* SES groups. It is clear though, that most of the studies on SES and 2009 pandemic not controlling for medical at risk factors [13 of 21], showed that lower SES groups have the highest risks of the three considered pandemic outcomes.

### Quantitative synthesis

**Fig 1:**
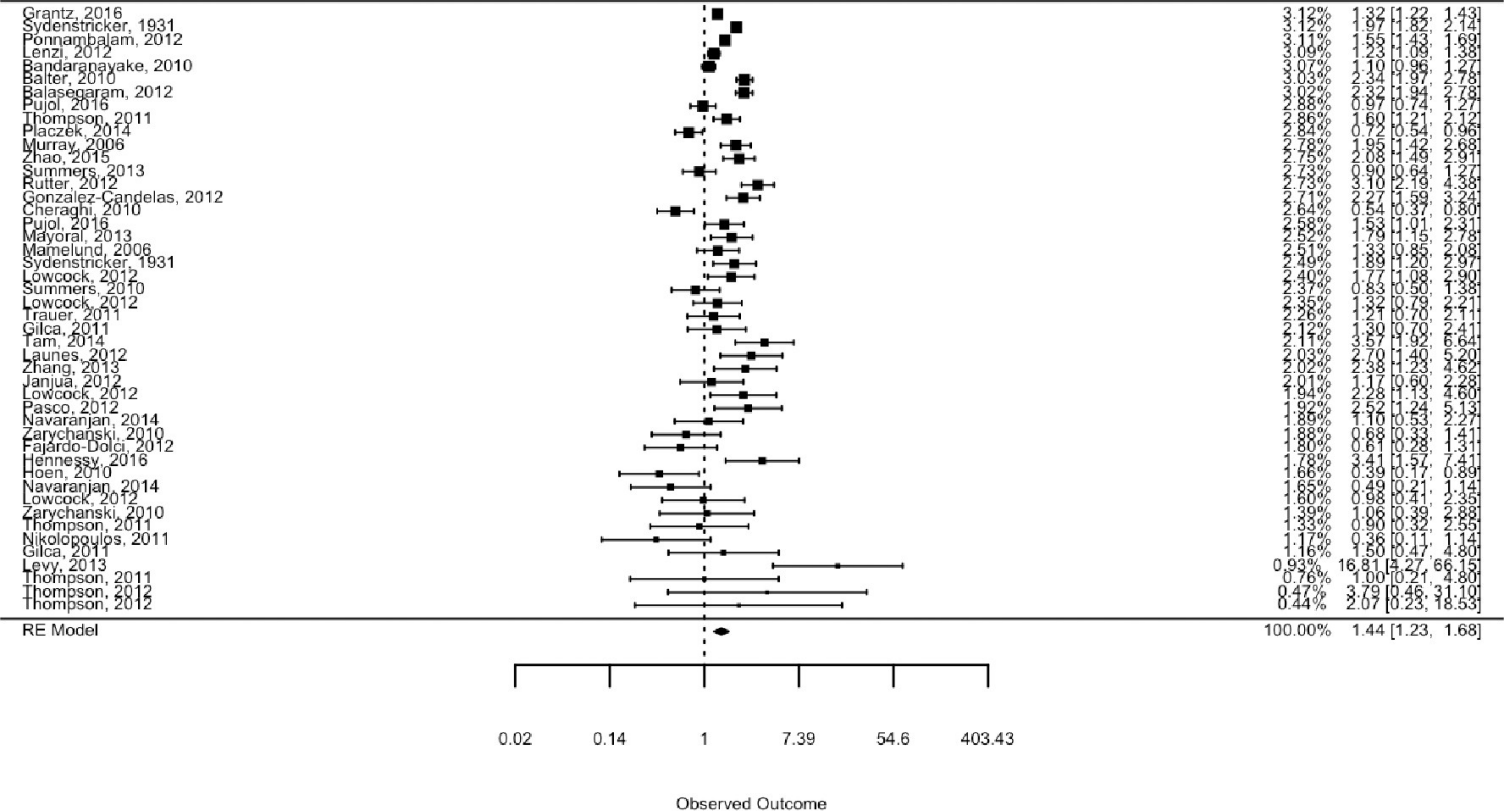
Forest plot. The plot shows the included estimates sorted by precision, along with their weights in the pooled effect estimate.

The quantitative synthesis includes 46 estimates drawn from 35 of the 44 studies included in the narrative synthesis [13–16, 27–30, 32–35, 37–41, 45–49, 51–57, 59–64], and a standard random effects analysis of all estimates pooled found a pooled effect mean odds ratio of 1.4 (95% CI: 1.2–1.7), comparing the low to the high SES groups. The pooled estimate was statistically significant at the 0.1 percent level, which means that we would have been highly unlikely to see an estimate of this or larger absolute magnitude if the true mean of the effect distribution was zero. As seen in the forest plot, the individual study estimates differed in both precision and location, with more variation in less precise estimates as we would expect (Fig 1). To test for the presence of publication bias, we used Egger’s test and Begg’s test as implemented in the regtest and ranktest commands of the Metafor R-package [23].As imprecise estimates have to be larger to be statistically significant, publication bias will tend to show up as a systematic relationship between point estimates and their standard errors. Neither a rank-rank correlation test (Begg’s test, p-value 0.68) nor a regression test (Egger’s test, p-value 0.81) indicated any such relationship.

**Fig 2:**
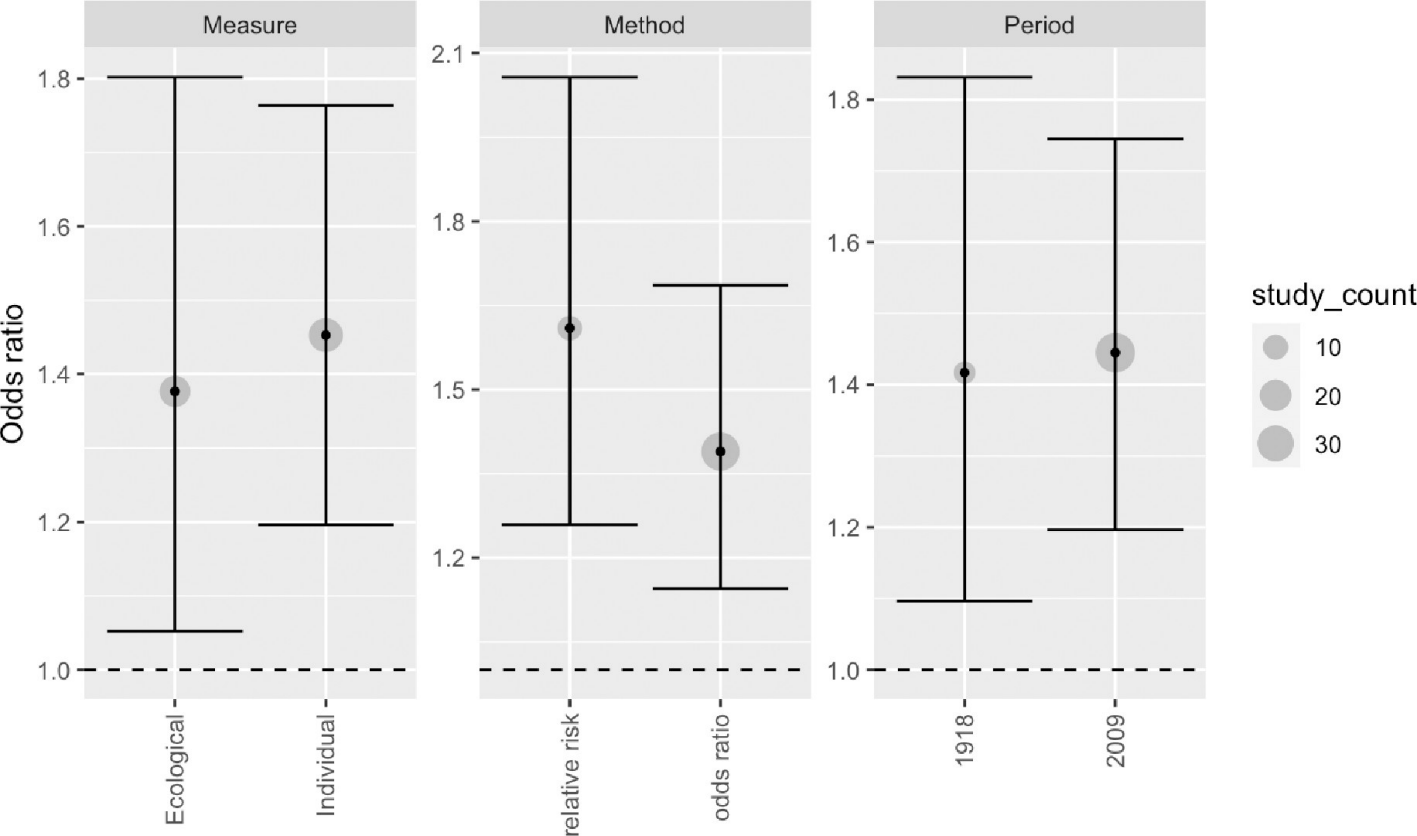
Subsample analyses. The plot shows point estimates and 95% confidence intervals for different subsamples of studies, with a grey circle indicating the number of studies in each subsample.

In the random effect analysis we found strong evidence of effect heterogeneity across studies, with an estimated 92% of the total variation across studies reflecting effect differences rather than sampling variation. The estimated standard deviation of the effect distribution (tau) has a point estimate of 0.45 on the log scale. If the underlying effects at the study level are normally distributed around their expectation, this tau is the estimated standard deviation of study effects. The estimates would then imply that there is a 50% chance that the true parameter value of a randomly selected study lies in the range of 1.1–1.9. The Cochran’s Q test strongly rejects a test of zero heterogeneity (p < 0.0001), confirming the choice of a random effects over a fixed effect model. Our subsample analyses indicate similar results in studies using individual level and aggregate SES indicators, case control and relative risk outcome measures, and studying the 1918 and 2009 pandemic period (Fig 2 and Table 2).

**Table 2:** Subsample analyses.

Distinction	Type	Number of estimates	Pooled RE effect	95% CI lower bound	95% CI upper bound	Tau
Measure	Ecological	20	1.38	1.05	1.80	0.53
	Individual	26	1.45	1.20	1.76	0.41
Period	1918	7	1.42	1.10	1.83	0.30
	2009	39	1.44	1.20	1.76	0.50
Method	Relative Risk	10	1.61	1.26	2.06	0.35
	Odds Ratio	36	1.39	1.14	1.69	0.49

The plot shows point estimates and 95% confidence intervals for different subsamples of studies, with a grey circle indicating the number of studies in each subsample.

**Fig 3:**
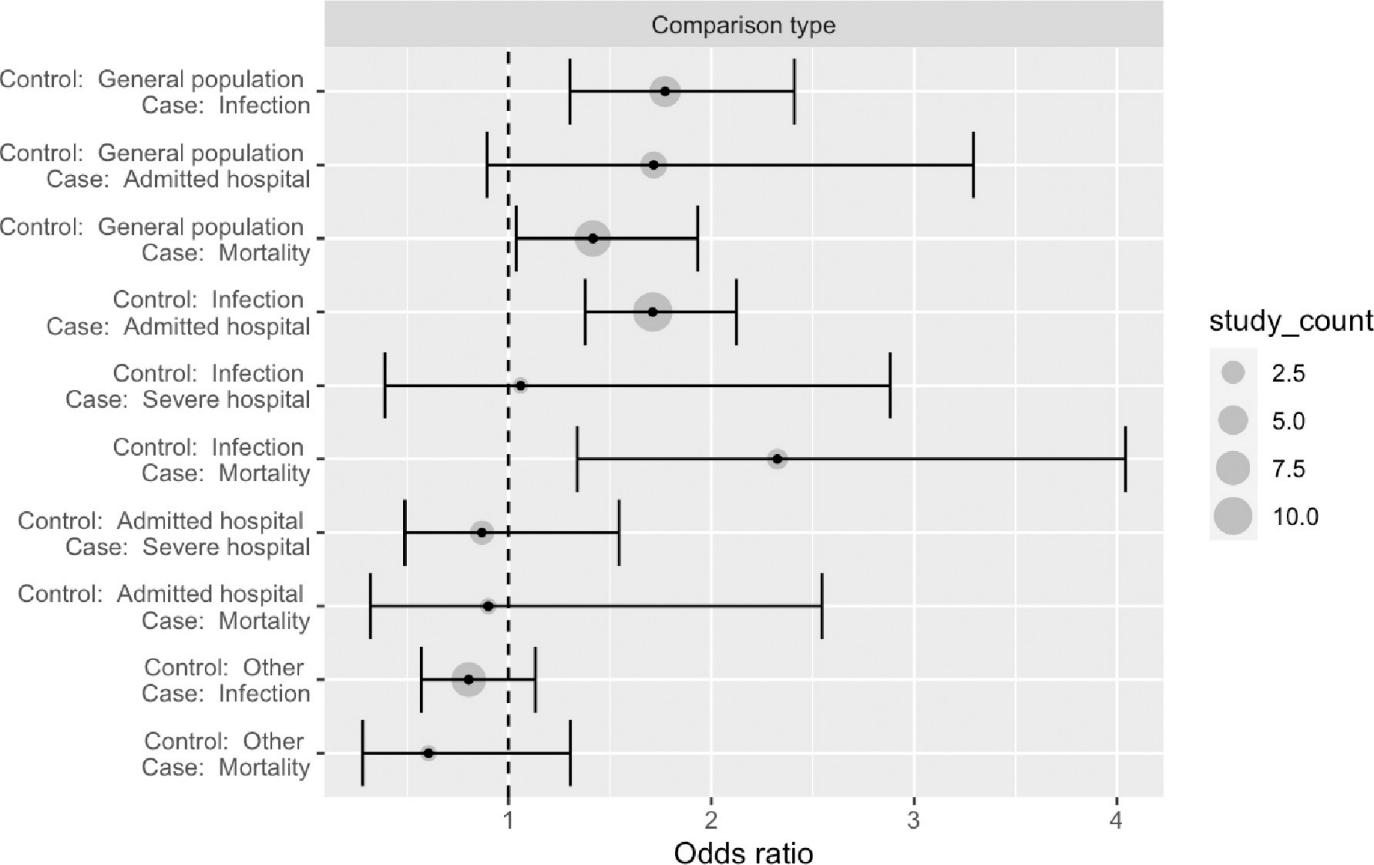
Subsample analyses. The plot shows point estimates and 95% confidence intervals for different subsamples of studies, with a grey circle indicating the number of studies in each subsample.

Subsamples were also defined by specific *combinations* of case and control outcomes (Fig 3). These suggest that studies examining the risk of flu outcomes relative to a general healthy population (here defined as a control sample not selected on indicators of illness) tend to indicate a clear and substantial increased risk for lower SES groups. Studies comparing hospitalized to those infected also point to increased risks for lower SES groups. Studies assessing the risk of severe cases (e.g., treatment in ICU or death) *conditional* on hospitalization are fewer, but seem to report no clear SES associations in any direction. Finally, studies using “other” control samples (e.g., patients with flu symptoms who did not have flu, people with non-pandemic flu during a pandemic period, patients accessing or being treated by health care systems for other reasons) tend to find no (or reversed) associations with SES indicators.

As all of these comparisons were based on different splits of the same study sample, they can be viewed as a series of univariate analyses. To assess the joint contribution of these study level features, and to include country/region indicators, we estimated two Bayesian models: One, without study level covariates, was closely analogous to the above meta-analysis, and was included to ensure that results from the two approaches were similar and comparable. This Bayesian model finds a pooled effect mean of 1.4 with a 95% credibility interval from 1.2–1.7, which is identical to the above estimate of 1.4 (95% CI: 1.2–1.7). The estimated standard deviation of the underlying study parameters, analogous to the parameter tau in the earlier analysis, is estimated at 0.46 (0.3–0.6), the same as the above estimated tau of 0.45 (See S2 File for model code and discussion of prior choices). The second Bayesian model includes all study level indicators (level of SES indicator, RR/OR indicator, period, case and control outcomes, and country/region), as well as an indicator for each unique *combination* of case and control outcome (as in Fig 3). Jointly, this reduces the estimated unexplained heterogeneity (tau) substantially, with the average value estimated dropping from 0.46 to 0.34 (See S2 File for model code and discussion of prior choices).

**Fig 4:**
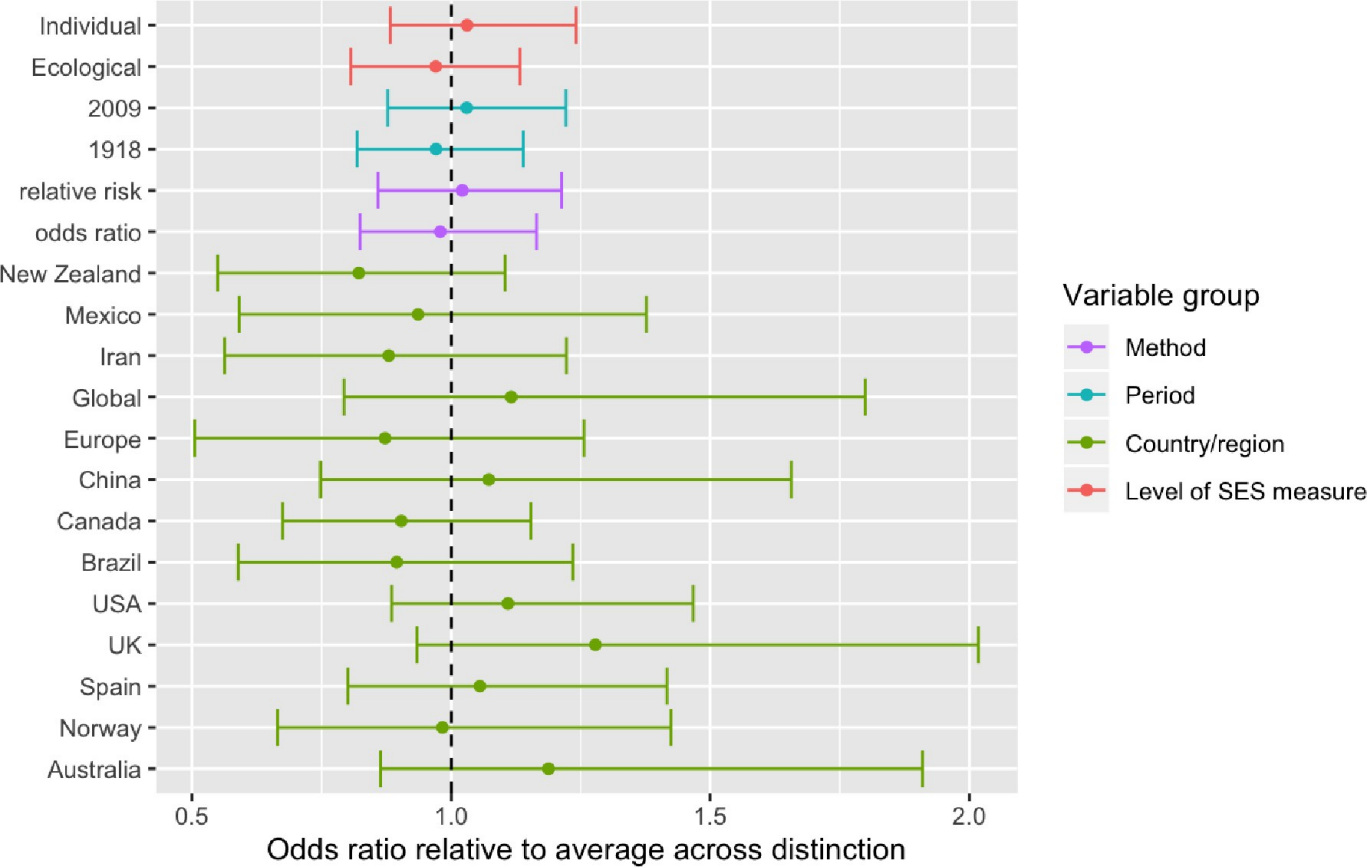
Differences across study level covariates. The plot shows average estimates and 95% credibility intervals for different study level covariates. The parameters are constrained to sum to zero within each category (e.g., for each draw from the posterior distribution, the sum of country parameters will sum to zero, as will the sum of the period parameters, etc.) See S2 File for model details.

**Fig 5:**
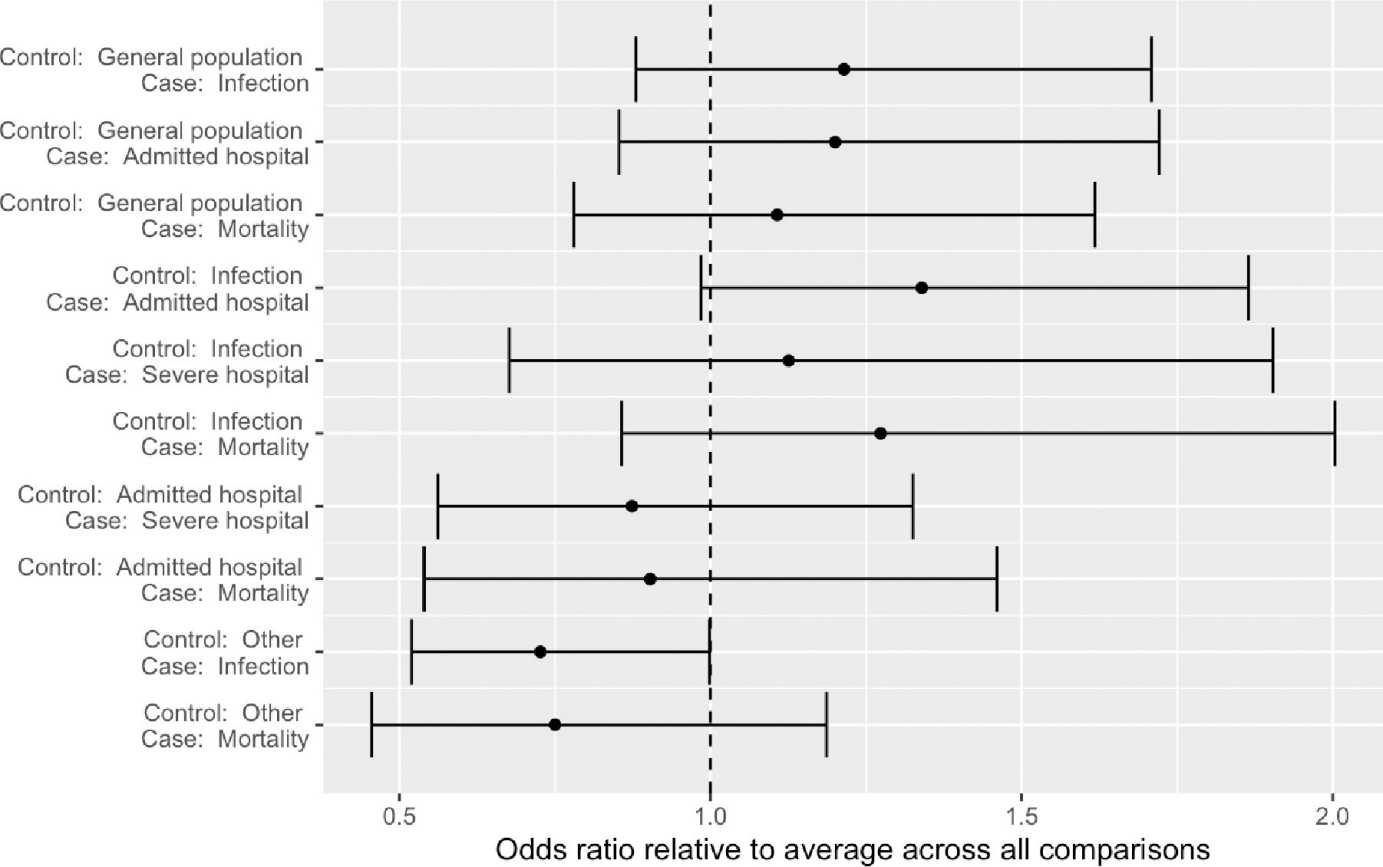
Differences across case and control outcome combinations. The plot shows average estimates and 95% credibility intervals for all combinations of case and control outcomes observed in the data. See S2 File for model details.

As shown in Figs 4 and 5, the Bayesian analysis finds similar results as the earlier subsample analyses, indicating that the patterns for the control and treatment outcome combinations are not “explained away” in an analysis when simultaneously accounting for other study level characteristics.

## Discussion

Research on Covid-19 has shown that the disease burden differs by SES, race and ethnicity [2–5]. This is consistent with the results we report here from the first systematic literature review on the associations between SES and disease outcomes in the last 5 influenza pandemics. We identified nine studies of the “Spanish flu of 1918–20” and 35 of the “Swine flu of 2009–2010”, but no studies of the “Russian flu” pandemic of 1889–90, the “Asian flu” of 1957–58 or the “Hong-Kong flu” of 1968–70. Most of the studies included for the 1918 and 2009 influenza pandemics used data from western high-income countries. Out of 51 estimates from 35 studies, the overall pooled mean pandemic outcome odds ratio was 1.44 (95% CI: 1.23–1.68) comparing the lowest to the highest SES groups. As expected, the random effect model estimated substantial effect heterogeneity across studies, which means the pooled effect mean should not be taken as valid at the single-study level. Based on the model, we would expect about 50% of the underlying effects to be in the 1.1 to 1.9 range. There was no evidence suggesting differences by pandemic period (1918 or 2009), the level of SES measure (individual or ecological), or type of method (odds ratio or relative risk). Finally, studies using healthy controls tended to find low SES associated with worse influenza outcome, and studies using infected controls found low SES associated with more severe influenza outcomes. Studies comparing severe outcomes (ICU or death) to hospital admissions were few but indicated no clear association. Studies with more unusual comparisons (e.g., pandemic vs seasonal influenza, seasonal influenza vs other patient groups) reported no or negative associations. These patterns were similar in a multivariate Bayesian model accounting for all study level indicators simultaneously. The Bayesian model also included indicators for study region/country. Relative to the “across all country/regions” average, studies from Australia, UK and to a lesser extent the USA tended to report stronger associations in our sample, while New Zealand tended to report weaker associations. These country-level results should be viewed as exploratory: two of the three studies from New Zealand [28, 29], for instance, were studies of how pandemic influenza outcomes varied across pre-service occupational status amongst military personnel during the 1918 pandemic, which are unlikely to speak broadly to such associations in New Zealand more generally.

Our results provide strong evidence that social risk factors matter for pandemic influenza outcomes in addition to medical risk factors. We also documented that in the 2009 pandemic, social risk factors independently explained variation in disease outcomes even when medical risk factors were controlled for [34, 46–48, 51, 54, 57, 59]. This is similar to the finding of a study of COVID-19 hospital deaths demonstrating that medical risk factors did little to explain the higher risks of the deprived and of immigrants in the UK [3]. Although we did not find support for our hypothesis that social disparities would be larger for more severe (e.g. ICU and death) than less severe outcomes (e.g. infection or hospitalization not requiring ICU), the similarity of results for the 1918 and 2009 pandemics show the persistence of individual- and ecological-level social risk factors, although the specific mechanisms and types of social vulnerabilities leading to social disparities in pandemic outcomes may differ between 1918 and 2009, or in 2020 during the COVID-19 pandemic. Results from this review on pandemic influenza and results from studies on the role of social and ethnic vulnerability in COVID-19 disease outcomes [2–5], support recent calls for the inclusion of social and ethnic vulnerabilities in addition to medical at risk factors in pandemic preparedness plans [18]. Examples are the prioritization of vaccines for medically vulnerable people living in socially vulnerable areas (urban slums or hard-to-reach groups in rural and remote areas), or SES groups with undiscovered medical vulnerabilities, and others who are at significantly higher risk of severe disease or death (various indigenous, ethnic, or racial groups, people living in extreme poverty, homeless and those living in informal settlements or; low-income migrant workers; refugees, internally displaced persons, asylum seekers, populations in conflict settings or those affected by humanitarian emergencies, vulnerable migrants in irregular situations and nomadic populations).

The studies reporting on social inequalities in influenza outcomes in 1918 and in 2009, identified in this review, and also early research on social disparities in COVID-19 outcomes, often lacked a discussion of the possible mechanisms for the estimated social disparities, a framework to discuss those mechanisms and/or the data to separate the distal (social and policy) and proximal (behavioral and biological factors) factors for unequal exposure, susceptibility and access to health care leading to socially unequal pandemic outcomes [68]. Socially unequal exposure may relate to hand washing behavior or mask use, cleaning of surfaces, cramped living conditions, multigenerational living, occupational exposure, ability to work from home or stay away from work to care for family members, and use of public transportation. Social disparities in susceptibility may relate to poor nutritional status or, concurrent illnesses (e.g. NCDs). Finally, socioeconomic inequalities in understanding of or access to health advice (e.g. hand hygiene, social distancing, travel advisories) and vaccination or other public recommendations due to poor reading and writing skills may also explain part of the variation in outcomes by SES [13, 18].

Two of the studies on the 2009 pandemic included in our review, on Iran [35] and USA [55], reported increased risks (of infection rates in Iran and ICU stay vs. hospitalized non-ICU patients in USA) for those with high socioeconomic status–contrary to the authors’ and our hypothesis. For the US study, the authors suggest that this may reflect social gradients in testing and demand for treatment and health care resources. In one matched case–control study of mortality among hospitalized H1N1 patients vs. H1N1 outpatients, no differences in any of the SES variables were found when controlling for health seeking behavior and barriers to health care access. However, it is not clear whether these or other controls (age, sex, race, urban-rural, vaccination status, health behaviors, pre-existing conditions) “explained away” the negative associations of having health care insurance or the positive association of poverty in the univariate models. If the studies with data generated from health care systems that found higher pandemic risks for lower SES groups had controlled for a social gradient in testing and demand for treatment and health care resources, we expect that the findings of a social pattern in disease burden would be reinforced.

An important strength of our study is the use of a pre-registered study protocol for data gathering and analysis, which was peer-reviewed and published prior to the gathering of study data [22]. This helped ensure that the process was specified in a reproducible way and followed a rigorous and systematic workflow to identify studies and describe and analyze results. The engagement of professional information specialists to design, test and improve the literature search strategies that were applied to a broad range of literature databases is particularly important, given the lack of any previous systematic reviews on this topic with which our list of included studies could be compared.

Our study also has some potential limitations. First, we carried out our library search on 17 November 2017, and potential studies published in the two pre-COVID-19 years of 2018–19 and during the COVID-19 pandemic (2020–21) are not included. Given the massive research on COVID-19 pandemic in 2020–21 and the fact that the identified 2009 pandemic studies in our review were published rapidly after 2009–10, with 2012 being the average publication year, it is likely that we have missed a few 2009 pandemic studies. Given the strength and consistency of the results, we do not expect that newer studies would alter our general conclusions, at least not for the 2009 pandemic that was the topic of 35 of the 44 included studies. Second, we would note that the generalizability of our results is necessarily limited by the geographic focus of the research we synthesize: no studies using data from Africa were found, and few from Asia and South America. It is therefore reasonable to ask whether our results are representative outside of high-income countries in North America, Europe and the Oceania region.

## Conclusion

We have shown that influenza pandemic outcomes in 1918 and 2009 were associated with lower socioeconomic status and that pandemic outcomes in 2009 were not always socially neutral «great equalizers» once adjusting for medical risk factors [34, 46–48, 51, 54, 57, 59]. This resembles the finding of a study of COVID-19 hospital deaths demonstrating that medical risk factors did little to explain the higher risks of the deprived and of immigrants in the UK [69]. The social lessons from historical influenza pandemics such as those in 1918 or 2009 have not yet been taken into account in influenza pandemic preparedness [18], and this blind spot has also been evident in the response to the COVID-19 pandemic. Such social and ethnic vulnerability factors should be explicitly included and addressed in current and future plans and responses in order to more effectively reduce pandemic burdens, reduce social disparities and ameliorate the social consequences of future pandemics [70]. The global health and economic crisis created by the COVID-19 pandemic has made us only too aware of the need for a more holistic and comprehensive approach towards pandemic preparedness.

## Supporting information

S1 Checklist PRISMA 2020 checklist. (PDF)

S1 File PRISMA flow diagram. (PDF)

S2 File Specific studies included and all judgments and adjustments concerning inclusion and adjustments of reported numbers. (PDF)

S1 Table Medline search strategy. (PDF)

S2 Table Quality assessments. (PDF)

## References

[1] Smith E. Madonna calls coronavirus ‘the great equalizer’ in bizarre bathtub video. Page Six. 2020 22 March.

[2] Steyn N, Binny R, Hannah K, Hendy S, James A, Kukutai T, et al. Estimated inequities in COVID-19 infection fatality rates by ethnicity for Aotearoa New Zealand. New Zealand Medical Journal. 2020;133(1520). 32994635

[3] Williamson EJ, Walker AJ, Bhaskaran K, Bacon S, Bates C, Morton CE, et al. Factors associated with COVID-19-related death using OpenSAFELY. Nature. 2020;584(7821):430–6. doi: 10.1038/s41586-020-2521-4 32640463 PMC7611074

[4] Drefahl S, Wallace M, Mussino E, Aradhya S, Kolk M, Brandén M, et al. A population-based cohort study of socio-demographic risk factors for COVID-19 deaths in Sweden. Nature Communications. 2020;11(1):5097. doi: 10.1038/s41467-020-18926-3 33037218 PMC7547672

[5] Liu SH, Liu B, Li Y, Norbury A. Time courses of COVID-19 infection and local variation in socioeconomic and health disparities in England. medRxiv. 2020:2020.05.29.20116921.

[6] Rice G. Black November. The 1918 influenza epidemic in New Zealand. Wellington, New Zealand: Allen and Unwin; 1988.

[7] Phillips H. Black October: The impact of the Spanish influenza epidemic of 1918 on South Africa: University of Cape Town; 1984.

[8] Tomkins SM. The Failure of Expertise: Public Health Policy in Britain during the 1918–19 Influenza Epidemic. Social History of Medicine. 1992;5(3):435–54. doi: 10.1093/shm/5.3.435 11623115

[9] Van Hartesveldt FR. The 1918–1919 pandemic of influenza: the urban impact in the western world. New York: Edwin Mellen Press; 1992.

[10] Crosby A. Epidemic and peace, 1918. Westport, Connecticut: Greenwood Press; 1976.

[11] Mamelund SE. Profiling a Pandemic. Who were the victims of the Spanish flu? Natural History Magazine. 2017(September):6–10.

[12] Mamelund S-E. Spanish Influenza Mortality of Ethnic Minorities in Norway 1918–1919. European Journal of Population / Revue européenne de Démographie. 2003;19(1):83–102.

[13] Mamelund S-E. A socially neutral disease? Individual social class, household wealth and mortality from Spanish influenza in two socially contrasting parishes in Kristiania 1918–19. Social Science & Medicine. 2006;62(4):923–40. doi: 10.1016/j.socscimed.2005.06.051 16084634

[14] Murray CJ, Lopez AD, Chin B, Feehan D, Hill KH. Estimation of potential global pandemic influenza mortality on the basis of vital registry data from the 1918–20 pandemic: a quantitative analysis. The Lancet. 2007;368(9554):2211–8. 10.1016/S0140-6736(06)69895-4 17189032

[15] Rutter PD, Mytton OT, Mak M, Donaldson LJ. Socio-economic disparities in mortality due to pandemic influenza in England. International Journal Of Public Health. 2012;57(4):745–50. doi: 10.1007/s00038-012-0337-1 22297400

[16] Tam K, Yousey-Hindes K, Hadler JL. Influenza-related hospitalization of adults associated with low census tract socioeconomic status and female sex in New Haven County, Connecticut, 2007–2011. Influenza and Other Respiratory Viruses. 2014;8(3):274–81. doi: 10.1111/irv.12231 24382111 PMC4181475

[17] Chandrasekhar R, Sloan C, Mitchel E, Ndi D, Alden N, Thomas A, et al. Social determinants of influenza hospitalization in the United States. Influenza & Other Respiratory Viruses. 2017;05:05. doi: 10.1111/irv.12483 28872776 PMC5720587

[18] Mamelund SE. Social Inequality–a Forgotten Factor in Pandemic Influenza Preparedness. Journal of the Norwegian Medical Association. 2017(12–13):911–3. doi: 10.4045/tidsskr.17.0273 28655260

[19] Tricco AC, Lillie E, Soobiah C, Perrier L, Straus SE. Impact of H1N1 on Socially Disadvantaged Populations: Systematic Review. PLoS ONE. 2012;7(6):1–17. doi: 10.1371/journal.pone.0039437 22761796 PMC3382581

[20] McGowan J, Sampson M, Salzwedel DM, Cogo E, Foerster V, Lefebvre C. PRESS Peer Review of Electronic Search Strategies: 2015 Guideline Statement. Journal of Clinical Epidemiology. 2016;75:40–6. doi: 10.1016/j.jclinepi.2016.01.021 27005575

[21] Wells GA, Shea B, Peterson J, Welch V, Losos M, P T. The Newcastle-Ottawa Scale (NOS) for assessing the quality of nonrandomised studies in meta-analyses.

[22] Mamelund S-E, Shelley-Egan C, Rogeberg O. The association between socioeconomic status and pandemic influenza: protocol for a systematic review and meta-analysis. Systematic Reviews. 2019;8(1):5. doi: 10.1186/s13643-018-0931-2 30609940 PMC6318944

[23] Viechtbauer W. Conducting Meta-Analyses in R with the metafor Package. 2010. 2010;36(3):48.

[24] Alinaghi N, Reed WR. Meta-analysis and publication bias: How well does the FAT-PET-PEESE procedure work? Res Synth Methods. 2018;9(2):285–311. doi: 10.1002/jrsm.1298 29532634

[25] Gelman A, Hill J, Yajima M. Why We (Usually) Don’t Have to Worry About Multiple Comparisons. Journal of Research on Educational Effectiveness. 2012;5(2):189–211.

[26] Chowell G, Bettencourt LM, Johnson N, Alonso WJ, Viboud C. The 1918–1919 influenza pandemic in England and Wales: spatial patterns in transmissibility and mortality impact. Proceedings of the Royal Society of London—Series B: Biological Sciences. 2008;275(1634):501–9. doi: 10.1098/rspb.2007.1477 18156123 PMC2596813

[27] Grantz KH, Cummings DAT, Glass GE, Rane MS, Salje H, Schachterle SE. Disparities in influenza mortality and transmission related to sociodemographic factors within Chicago in the pandemic of 1918. Proceedings of the National Academy of Sciences of the United States of America. 2016;113(48):13839–44. doi: 10.1073/pnas.1612838113 27872284 PMC5137773

[28] Summers JA, Shanks GD, Baker MG, Wilson N. Severe impact of the 1918–19 pandemic influenza in a national military force. The New Zealand Medical Journal. 2013;126(1378):36–47. 24045314

[29] Summers JA, Wilson N, Baker MG, Shanks GD. Mortality risk factors for pandemic influenza on New Zealand troop ship, 1918. Emerg Infect Dis. 2010;16(12):1931–7. doi: 10.3201/eid1612.100429 21122224 PMC3294590

[30] Sydenstricker E. The Incidence of Influenza among Persons of Different Economic Status during the Epidemic of 1918. Public Health Reports (1896–1970). 1931;46(4):154–70. 16550779

[31] Pearce DC, Pallaghy PK, McCaw JM, McVernon J, Mathews JD. Understanding mortality in the 1918–1919 influenza pandemic in England and Wales. Influenza & Other Respiratory Viruses. 2011;5(2):89–98. doi: 10.1111/j.1750-2659.2010.00186.x 21306572 PMC4954464

[32] Balasegaram S, Ogilvie F, Glasswell A, Anderson C, Cleary V, Turbitt D, et al. Patterns of early transmission of pandemic influenza in London—link with deprivation. Influenza & Other Respiratory Viruses. 2012;6(3):e35–41. doi: 10.1111/j.1750-2659.2011.00327.x 22236079 PMC4941677

[33] Balter S, Gupta LS, Lim S, Fu J, Perlman SE. Pandemic (H1N1) 2009 surveillance for severe illness and response, New York, New York, USA, April-July 2009. Emerging Infectious Diseases. 2010;16(8):1259–64. doi: 10.3201/eid1608.091847 20678320 PMC3298321

[34] Bandaranayake D, Huang QS, Bissielo A, Wood T, Mackereth G, Baker MG, et al. Risk factors and immunity in a nationally representative population following the 2009 influenza A(H1N1) pandemic. PLoS ONE [Electronic Resource]. 2010;5(10):e13211. doi: 10.1371/journal.pone.0013211 20976224 PMC2954793

[35] Cheraghi Z, Irani AD, Rezaiean S, Ahmadzadeh J, Poorolajal J, Erfani H, et al. Influenza A (H1N1) in Hamedan Province, Western Iran in 2009: A case-control study. Journal of Research in Health Sciences. 2010;10(1):15–21. 22911912

[36] Duggal A, Pinto R, Rubenfeld G, Fowler RA. Global Variability in Reported Mortality for Critical Illness during the 2009–10 Influenza A(H1N1) Pandemic: A Systematic Review and Meta-Regression to Guide Reporting of Outcomes during Disease Outbreaks. PLoS ONE. 2016;11(5):1–14. doi: 10.1371/journal.pone.0155044 27170999 PMC4865181

[37] Fajardo-Dolci G, Gutierrez JP, Arboleya-Casanova H, Garcia-Saiso S. Comparing Deaths from Influenza H1N1 and Seasonal Influenza A:Main Sociodemographic and Clinical Differences between the Most Prevalent 2009 Viruses. Influenza Research & Treatment. 2012:1–5. doi: 10.1155/2012/501784 23346393 PMC3546448

[38] Gilca R, de Serres G, Boulianne N, Ouhoummane N, Papenburg J, Douville-Fradet M, et al. Risk factors for hospitalization and severe outcomes of 2009 pandemic H1N1 influenza in Quebec, Canada. Influenza and other Respiratory Viruses. 2011;5(4):247–55. doi: 10.1111/j.1750-2659.2011.00204.x 21651735 PMC4634547

[39] González-Candelas F, Astray J, Alonso J, Castro A, Cantón R, Galán JC, et al. Sociodemographic Factors and Clinical Conditions Associated to Hospitalization in Influenza A (H1N1) 2009 Virus Infected Patients in Spain, 2009–2010. PLoS ONE. 2012;7(3):1–8. 10.1371/journal.pone.0033139 PMC3296770 22412995

[40] Hennessy TW, Bruden D, Castrodale L, Komatsu K, Erhart LM, Thompson D, et al. A case-control study of risk factors for death from 2009 pandemic influenza A(H1N1): is American Indian racial status an independent risk factor? Epidemiology And Infection. 2016;144(2):315–24. doi: 10.1017/S0950268815001211 26118767 PMC5222627

[41] Hoen AG, Buckeridge DL, Chan EH, Freifeld CC, Keller M, Charland K, et al. Characteristics of US public schools with reported cases of novel influenza A (H1N1). International Journal of Infectious Diseases. 2010;14 Suppl 3:e6-8. 10.1016/j.ijid.2009.11.034 PMC2914813 20363169

[42] Hu W, Williams G, Phung H, Birrell F, Tong S, Mengersen K, et al. Did socio-ecological factors drive the spatiotemporal patterns of pandemic influenza A (H1N1)? Environment International. 2012;45(Supplement C):39–43. doi: 10.1016/j.envint.2012.03.010 22572115

[43] Huang X, Clements AC, Williams G, Mengersen K, Tong S, Hu W. Bayesian estimation of the dynamics of pandemic (H1N1) 2009 influenza transmission in Queensland: A space-time SIR-based model. Environmental Research. 2016;146:308–14. doi: 10.1016/j.envres.2016.01.013 26799511

[44] Inglis NJ, Bagnall H, Janmohamed K, Suleman S, Awofisayo A, De Souza V, et al. Measuring the effect of influenza A(H1N1)pdm09: the epidemiological experience in the West Midlands, England during the ’containment’ phase. Epidemiology & Infection. 2014;142(2):428–37. 23731730 10.1017/S0950268813001234 PMC9151159

[45] Janjua NZ, Skowronski DM, Hottes TS, Osei W, Adams E, Petric M, et al. Transmission dynamics and risk factors for pandemic H1N1-related illness: outbreak investigation in a rural community of British Columbia, Canada. Influenza & Other Respiratory Viruses. 2012;6(3):e54–e62. 22385647 10.1111/j.1750-2659.2012.00344.x PMC4986582

[46] Launes C, García-García JJ, Martínez-Planas A, Moraga F, Astigarraga I, Arístegui J, et al. 2009 H1N1: risk factors for hospitalization in a matched case-control study. European Journal of Pediatrics. 2012;171(7):1127–31. doi: 10.1007/s00431-012-1716-6 22430351

[47] Lenzi L, Mello AM, Silva LR, Grochocki MH, Pontarolo R. Pandemic influenza A (H1N1) 2009: risk factors for hospitalization. Jornal Brasileiro De Pneumologia: Publicacao Oficial Da Sociedade Brasileira De Pneumologia E Tisilogia. 2012;38(1):57–65. doi: 10.1590/s1806-37132012000100009 22407041

[48] Levy NS, Nguyen TQ, Westheimer E, Layton M. Disparities in the Severity of Influenza Illness: A Descriptive Study of Hospitalized and Nonhospitalized Novel H1N1 Influenza-Positive Patients in New York City: 2009–2010 Influenza Season. Journal of Public Health Management & Practice. 2013;19(1):16–24. doi: 10.1097/PHH.0b013e31824155a2 23169399

[49] Lowcock EC, Rosella LC, Foisy J, McGeer A, Crowcroft N. The Social Determinants of Health and Pandemic H1N1 2009 Influenza Severity. American Journal of Public Health. 2012;102(8):e51–e8. doi: 10.2105/AJPH.2012.300814 22698024 PMC3464856

[50] Maliszewski PJ, Wei R. Ecological factors associated with pandemic influenza A (H1N1) hospitalization rates in California, USA: a geospatial analysis. Geospatial Health. 2011;6(1):95–105. doi: 10.4081/gh.2011.161 22109867

[51] Mayoral JM, Alonso J, Garín O, Herrador Z, Astray J, Baricot M, et al. Social factors related to the clinical severity of influenza cases in Spain during the A (H1N1) 2009 virus pandemic. BMC Public Health. 2013;13(1):1–7. doi: 10.1186/1471-2458-13-118 23391376 PMC3572414

[52] Navaranjan D, Rosella LC, Kwong JC, Campitelli M, Crowcroft N. Ethnic disparities in acquiring 2009 pandemic H1N1 influenza: a case–control study. BMC Public Health. 2014;14(1):1–17. doi: 10.1186/1471-2458-14-214 24580862 PMC3942768

[53] Nikolopoulos G, Bagos P, Lytras T, Bonovas S. An Ecological Study of the Determinants of Differences in 2009 Pandemic Influenza Mortality Rates between Countries in Europe. PLoS ONE. 2011;6(5):1–8. doi: 10.1371/journal.pone.0019432 21589928 PMC3092762

[54] Pasco JA, Nicholson GC, Brennan SL, Bennett KE, Dobbins AG, Athan E. The epidemiology of the first wave of H1N1 influenza pandemic in Australia: A population-based study. Open Public Health Journal. 2012;5:80–5.

[55] Placzek H, Madoff L. Effect of Race/Ethnicity and Socioeconomic Status on Pandemic H1N1-Related Outcomes in Massachusetts. American Journal of Public Health. 2014;104(1):e31–e8. doi: 10.2105/AJPH.2013.301626 24228651 PMC3910053

[56] Ponnambalam L, Samavedham L, Lee HR, Ho CS. Understanding the socioeconomic heterogeneity in healthcare in US counties: the effect of population density, education and poverty on H1N1 pandemic mortality. Epidemiology And Infection. 2012;140(5):803–13. doi: 10.1017/S0950268811001464 21835071

[57] Pujol J, Godoy P, Soldevila N, Castilla J, González-Candelas F, Mayoral JM, et al. Social class based on occupation is associated with hospitalization for A(H1N1)pdm09 infection. Comparison between hospitalized and ambulatory cases. Epidemiology And Infection. 2016;144(4):732–40. doi: 10.1017/S0950268815001892 26271901

[58] Simonsen L, Spreeuwenberg P, Lustig R, Taylor RJ, Fleming DM, Kroneman M, et al. Global mortality estimates for the 2009 Influenza Pandemic from the GLaMOR project: a modeling study. PLoS Medicine. 2013;10:e1001558–1277. doi: 10.1371/journal.pmed.1001558 24302890 PMC3841239

[59] Thompson DL, Jungk J, Hancock E, Smelser C, Landen M, Nichols M, et al. Risk Factors for 2009 Pandemic Influenza A (H1N1)-Related Hospitalization and Death Among Racial/Ethnic Groups in New Mexico. American Journal of Public Health. 2011;101(9):1776–84. doi: 10.2105/AJPH.2011.300223 21778495 PMC3154223

[60] Thompson LH, Mahmud SM, Keynan Y, Blanchard JF, Slater J, Dawood M, et al. Serological survey of the novel influenza A H1N1 in inner city Winnipeg, Manitoba, 2009. The Canadian Journal of Infectious Diseases & Medical Microbiology. 2012;23(2):65–70. 23730311 10.1155/2012/484693 PMC3403663

[61] Trauer JM, Laurie KL, McDonnell J, Kelso A, Markey PG. Differential effects of pandemic (H1N1) 2009 on remote and indigenous groups, Northern Territory, Australia, 2009. Emerging Infectious Diseases. 2011;17(9):1615–23. doi: 10.3201/eid1709.101196 21888786 PMC3322054

[62] Zarychanski R, Stuart TL, Kumar A, Doucette S, Elliott L, Kettner J, et al. Correlates of severe disease in patients with 2009 pandemic influenza (H1N1) virus infection. CMAJ Canadian Medical Association Journal. 2010;182(3):257–64. doi: 10.1503/cmaj.091884 20093297 PMC2826467

[63] Zhang Y, Seale H, Yang P, MacIntyre CR, Blackwell B, Tang S, et al. Factors associated with the transmission of pandemic (H1N1) 2009 among hospital healthcare workers in Beijing, China. Influenza & Other Respiratory Viruses. 2013;7(3):466–71. doi: 10.1111/irv.12025 23078163 PMC5779818

[64] Zhao H, Harris RJ, Ellis J, Pebody RG. Ethnicity, deprivation and mortality due to 2009 pandemic influenza A(H1N1) in England during the 2009/2010 pandemic and the first post-pandemic season. Epidemiology And Infection. 2015;143(16):3375–83. doi: 10.1017/S0950268815000576 25850904 PMC9150971

[65] Mamelund S-E, Dimka J. Tuberculosis as a Risk Factor for 1918 Influenza Pandemic Outcomes. Tropical Medicine and Infectious Disease. 2019;4(2):74. doi: 10.3390/tropicalmed4020074 31035651 PMC6630781

[66] Dimka J, Mamelund S-E. 1918 Influenza Outcomes among Institutionalized Norwegian Populations: Implications for Disability-Inclusive Pandemic Preparedness. Scandinavian Journal of Disability Research. 2020;22(1):175–86.

[67] Galobardes B, Shaw M, Lawlor DA, Lynch JW. Indicators of socioeconomic position (part 1). Journal of Epidemiology and Community Health. 2006;60(1):7–12. doi: 10.1136/jech.2004.023531 16361448 PMC2465546

[68] Quinn SC, Kumar S. Health inequalities and infectious disease epidemics: a challenge for global health security. Biosecurity And Bioterrorism: Biodefense Strategy, Practice, And Science. 2014;12(5):263–73. 10.1089/bsp.2014.0032 PMC4170985 25254915

[69] Williamson E, Walker AJ, Bhaskaran KJ, Bacon S, Bates C, Morton CE, et al. OpenSAFELY: factors associated with COVID-19-related hospital death in the linked electronic health records of 17 million adult NHS patients. medRxiv. 2020:2020.05.06.20092999.

[70] Schmidt H, Pathak P, Sönmez T, Ünver MU. Covid-19: how to prioritize worse-off populations in allocating safe and effective vaccines. BMJ. 2020;371:m3795. doi: 10.1136/bmj.m3795 33020072

