# Supplementary material for: The association between socioeconomic status and pandemic influenza: Systematic review and meta-analysis
Source: PLoS One. 2021 Sep 7;16(9):e0244346. doi: 10.1371/journal.pone.0244346 (PMC8423272; doi:10.1371/journal.pone.0244346)
Supplement: S1 Table — (PDF) [file pone.0244346.s004.pdf]

Medline search strategy

#	Searches
1	Orthomyxoviridae/
2	Influenzavirus A/
3	Influenza A virus/
4	Influenza A Virus, H1N1 Subtype/
5	Influenza A Virus, H1N2 Subtype/
6	Influenza A Virus, H2N2 Subtype/
7	Influenza A Virus, H3N2 Subtype/
8	Influenza A Virus, H3N8 Subtype/
9	Influenza Pandemic, 1918-1919/
10	Pandemics/
11	Disease Outbreaks/
12	1 or 2 or 3 or 4 or 5 or 6 or 7 or 8
13	10 or 11
14	12 and 13
15	((Influenza or flu) adj3 (pandemic* or outbreak* or epidemic*)).tw,kw,kf.
16	((Russian or Spanish or Asian or Hong Kong or Mexican) adj3 (flu or influenza or pandemic* or epidemic*)).tw,kw,kf.
17	((H1N1 or H1N2 or H2N2 or H3N2 or H3N8) adj3 (pandemic* or outbreak* or epidemic*)).tw,kw,kf.
18	(PH1N1 or H1N1pdm09 or H1N1p).tw,kw,kf.
19	(("1889" or 1889-90 or 1889-1890 or 1889-91 or 1889-1891 or 1889-92 or 1889-1892 or 1889-93 or 1889-1893 or 1889-94 or 1889-1894 or 1918-19 or 1918-1919 or 1918-20 or 1918-1920 or "1957" or "1958" or 1957-58 or 1957-1958 or "1968" or 1968-69 or 1968-1969 or 1968-70 or 1968-1970 or "1969" or 1969-70 or 1969-1970 or "1970" or "2009" or 2009-10 or 2009-2010) adj7 (flu or influenza or pandemic* or epidemic* or outbreak*)).tw,kw,kf.
20	9 or 14 or 15 or 16 or 17 or 18 or 19
21	Socioeconomic Factors/
22	Poverty/
23	Poverty Areas/
24	Working Poor/
25	Social Class/
26	Social Conditions/
27	Social Marginalization/
28	Social Isolation/
29	Educational Status/
30	Employment/

31	Medical Indigency/
32	Medically Uninsured/
33	"Social Determinants of Health"/
34	Vulnerable Populations/
35	Minority Groups/
36	"Transients and Migrants"/
37	Housing/
38	Crowding/
39	Indians, North American/
40	Indians, Central American/
41	Indians, South American/
42	Inuits/
43	Alaska Natives/
44	Oceanic Ancestry Group/
45	Refugees/
46	"Emigrants and Immigrants"/
47	"Emigration and Immigration"/
48	Ethnic Groups/
49	Demography/
50	(((social* or socioeconomic* or socio-economic* or sociodemographic* or socio-demographic* or socioecologic* or socio-ecologic* or economic*) adj3 (factor* or condition* or aspect* or impact* or indicator* or indice* or index* or disparit* or difference* or depriv* or inequalit* or justice or injustice or determin* or class* or status)) or SES).tw,kw,kf.
51	(Gini adj1 (coefficient or ratio or index)).tw,kw,kf.
52	(health adj3 (inequalit* or difference* or disparit*)).tw,kw,kf.
53	(living condition* or poverty or material deprivation* or crowding or housing or employment or unemployment or income* or wealth or social vulnerability or ((public or social) adj3 (welfare or security)) or working class or slum* or homelessness or homeownership or apartment size).tw,kw,kf.
54	((poor or vulnerable or underprivileged or disadvantaged or homeless or remote) adj3 (people or person* or group* or communit* or parish* or neighbourhood* or countr* or population* or area*)).tw,kw,kf.
55	(literacy or illiteracy or educat*).tw,kw,kf.
56	(Nativ* or immigrant* or refugee* or migrant* or minorit*).tw,kw,kf.
57	(indigenous or ethnic or American Indian* or Alaska native* or aborigin* or maori or inuit* or Yupik or Cupik or Inupiaq or Saint Lawrence Island Yupik or Unangax or Alutiiq or Eyak or Tlingit or Haida or Tsimshian or sami or eskimo* or Torres strait islander* or pacific islander* or pacific people or athabascan).tw,kw,kf.
58	or/21-57
59	20 and 58

60	limit 59 to (danish or english or norwegian or swedish)
