# Supplementary material for: The association between socioeconomic status and pandemic influenza: Systematic review and meta-analysis
Source: PLoS One. 2021 Sep 7;16(9):e0244346. doi: 10.1371/journal.pone.0244346 (PMC8423272; doi:10.1371/journal.pone.0244346)
Supplement: S2 Table — (PDF) [file pone.0244346.s005.pdf]

**Studies**	**Selection**		**Comparability**			**Data quality**		**Sum score**	**Included in the meta-analysis**
Studies included in the narrative synthesis *****	Broad and representative sample/pop of the exposed?<br>1=truly and somewhat<br>0=selected sub-groups and no description of pop.	Selection of non-exposed sample/pop<br>1=drawn from same community as the exposed<br>0=drawn from different source or no description	Confounder controls?<br>1=yes<br>0=no	Biological controls?<br>1=yes<br>0=no	SES Sig. beyond bio controls?<br>1=yes<br>0=no	Lab-confirmed outcome?<br>1=yes<br>0=no	Data aggregation level<br>1=individual<br>0=aggregate	**<u>44 studies</u>**:<br>average=4.5<br>median=4<br>min=2, max=7<br>***<u>35 studies incl. in meta – analysis:</u>***<br>average=4.9<br>median=5<br>min=3, max=7<br>**<u>9 studies not part of meta-analysis:</u>**<br>average=3.1<br>median=3<br>min=2, max=4	Yes=1<br>No=0
2	1	1	1	0		1	0	4	1
3	1	1	1	0		1	0	4	1
4	1	1	1	1	0	1	1	6	1
6	1	1	1	1	1	1	1	7	1
7	1	1	1	0		0	0	3	0
10	1	1	0			1	0	3	0
11	1	1	1			1	1	5	1
12	1	1	1	1	0	1	1	6	1
13	1	1	1	0		1	1	5	1
14	1	1	1	0		0	0	3	1
15	1	1	1	1	0	1	1	6	1
16	1	1	1	0		0	0	3	1
17	1	1	1	0		1	0	4	0
18	1	1	1	0		1	0	4	0
19	1	1	0			1	0	3	0
21	1	1	1	1	0	1	1	6	1
23	1	1	1	1	1	1	1	7	1
24	1	1	1	1	1	1	1	7	1
26	1	1	1	1	1	1	1	7	1
28	1	1	1			1	1	5	1
29	1	1	1	0		0	0	3	0
30	1	1	1	0		0	0	3	0
31	1	1	1	0		0	1	4	1
34	1	1	1	1	1	1	1	7	1
35	1	1	1			0	0	3	1
36	1	1	1	1	0	1	0	5	1
37	1	1	1	0		1	0	4	1
38	1	1	1	1	1	1	1	7	1
39	1	1	1	0		0	0	3	0
40	1	1	1	0		0	0	3	1
41	1	1	0	0		1	0	3	1
42	1	1	1	1	1	1	1	7	1
44	1	1	1	0		0	0	3	1
45	1	1				0	0	2	0
48	1	1	0	0		0	1	3	1
49	1	1	1	0		0	1	4	1
50	1	1	1	0		0	0	3	1
51	1	1	0	0		1	0	3	1
52	1	1	1	1	1	1	0	6	1
53	1	1	0	0		1	1	4	1
55	1	1	1			1	1	5	1
57	1	1	1	1	0	1	1	6	1
58	1	1	1	0		1	1	5	1
59	1	1	1	0		1	1	5	1

* These numbers correspond to the 59 studies from which we extracted data. In the data extraction phase, we removed an additional 15 studies The final number of studies included in the narrative synthesis was therefore the 44 listed in this table, also see documentation in supporting information file no. 4.
