# Supplementary material for: The association between socioeconomic status and pandemic influenza: Systematic review and meta-analysis
Source: PLoS One. 2021 Sep 7;16(9):e0244346. doi: 10.1371/journal.pone.0244346 (PMC8423272; doi:10.1371/journal.pone.0244346)
Supplement: Attachment — Submitted filename: REVIEWER COMMENTS.docx [file pone.0244346.s006.docx]

REVIEWER COMMENTS

The association between socioeconomic status and pandemic influenza: systematic
review and meta-analysis

Thank you for the opportunity to review this paper. I find the objective interesting, but it appears that this study has no significant addition to knowledge. Only 1918 and 2009 pandemics were included. Besides, a systematic review for 2009 pandemic has been published, and I find 1918 pandemic as obsolete to judge what happens in today’s global health system. There is a higher inequality and disparity in societies 100 years ago compared to 2009. So, a pooled effect of 1918 and 2009 possess some validity issues. Also, given the very high heterogeneity, the validity of the meta-analysis is questionable.

Generally, the presentation of this paper needs to be improved in terms of language, choice of words, punctuations, and special characters.

Here are some specific points below:

Abstract

1. Please check for use of past and present tense all through the paper.

E.g., “Results are summarized narratively and using meta-analytic strategies”.

2. “Studies comparing severe outcomes (ICU or death) to hospital admissions are few but indicate no clear association.”

“..Indicate no clear association” or “indicate no significant association”?

The term “clear” does not sound statistically right.

3. The conclusion was poorly written. They conclusion should be clear with a concise take-home message linked to the study objective – “whether and to what extent there was an
   association between socioeconomic status (SES) and disease outcomes in the last five
   influenza pandemics”. See the authors conclusion below:

*“Results show that social risk factors help to explain pandemic outcomes in 1918 and in 2009 although the mechanisms and types of social vulnerabilities leading to disparities in outcomes may differ over time. Studies of the 2009 pandemic also showed that social vulnerability could not always be explained by medical risk factors.”*

This should be in the discussion section of this paper. Also, there should be a full stop mark after 2009.

A good example of conclusion should be: “Socioeconomic status are not associated with pandemic influenza”. OR “There is no significant association between socioeconomic status and pandemic influenza”.

4. What is the significance level of the pooled estimate? This is important for a meta-analysis result but found to be lacking in the abstract.

Introduction

1. Page 3, Line 54 – 56: “A systematic assessment of the evidence for such risk inequalities has been lacking, however, apart from a systematic review and meta-analysis of how the risk of 2009 influenza pandemic outcomes differ for disadvantaged populations (mainly indigenous people) (20).”

I am not fully convinced of the addition to knowledge of this study. The authors already stated in the abstract that they only got data for the 1918 and 2009 pandemics. 1918 seems too obsolete (over a 100 years), which can affect the internal validity of their findings. Already, a metanalysis for the 2009 pandemic has been performed, so the addition of 1918 pandemic association with SES (which appears obsolete to this reviewer), questions the addition to knowledge of this paper.

Second, the above statement still needs to be rephrased: E.g., “apart from a systematic review and meta-analysis of how the risk of 2009 influenza pandemic outcomes differ for disadvantaged populations (mainly indigenous people) (20), a systematic assessment of the evidence for such risk inequalities has been lacking.

In summary of this point, I find no substantial addition to knowledge of this paper, in its current state.

2. Page 4, line 69 - 74: “Our review identified studies on the 1918 and 2009 pandemics only, with evidence of a social gradient in the disease burden of both these pandemics……….”.

It is wrong to place this statement in the introduction. The last paragraph of the introduction should be in the results section.

3. Materials and methods:

I found the search period to be obsolete. 2017 is not a recent search. What if recent studies in 2019 or 2020 exist that collected retrospective data from hospitals/agencies, and performed studies on the association of SES and influenza? How are the authors sure, some studies were not missed? I expect to see search up to at least June 2020.

4. Page 5, line 92: “However, we did not get any responses that made it into the paper and our analysis”

Authors should check for word choice here.

5. The inclusion and exclusion criteria are too lengthy. The authors mixed up inclusion criteria and search strategy. This needs to be clearly distinguished for ease of reading.
6. Page 6, line 135: “pilot tested”…

There should be a hyphen

7. Data synthesis is deficient. The content is poorly arranged. Some information does not need to be in this section but previous sections. Also, the authors should use reported speech. The writing style seems like a proposal of what they plan to do, when they have already completed the study.

The authors need to improve on their description of this section. It is structurally poor.

8. Page 37, line 373: Quantitative meta-analysis

All meta-analyses are quantitative. Better to say, “Quantitative synthesis”.

No information about the degree of heterogeneity that should prevent quantitative synthesis.
